# Four Prenylflavone Derivatives with Antiplasmodial Activities from the Stem of *Tephrosia purpurea* subsp. *leptostachya*

**DOI:** 10.3390/molecules22091514

**Published:** 2017-09-10

**Authors:** Yoseph Atilaw, Lois Muiva-Mutisya, Albert Ndakala, Hoseah M. Akala, Redemptah Yeda, Yu J. Wu, Paolo Coghi, Vincent K. W. Wong, Máté Erdélyi, Abiy Yenesew

**Affiliations:** 1 Department of Chemistry, University of Nairobi, Nairobi, P.O. Box 30197-00100, Kenya; gebreyos@gmail.com (Y.A.); loismwikali@yahoo.com (L.M.-M.); andakala@uonbi.ac.ke (A.N.); 2 Global Emerging Infections Surveillance (GEIS) Program, United States Army Medical Research Unit-Kenya (USAMRU-K), Kenya Medical Research Institute (KEMRI)—Walter Reed Project, Kisumu and Nairobi, P.O. Box 54-40100, Kisumu, Kenya; hoseaakala@yahoo.com (H.M.A.); redemptah.yeda@usamru-k.org (R.Y.); 3 State Key Laboratory of Quality Research in Chinese Medicine, Macau University of Science and Technology, Macau, P.O. Box 999078, China; yuki-ng92@hotmail.com (Y.J.W.); bowaiwong@gmail.com (V.K.W.W.); vecchioco@gmail.com (P.C.); 4 Department of Chemistry and Molecular Biology, University of Gothenburg, SE-40530 Gothenburg, Sweden; 5 Swedish NMR Centre, University of Gothenburg, P.O. Box 465, SE-40530 Gothenburg, Sweden

**Keywords:** *Tephrosia purpurea* subsp. *leptostachya*, stem, flavone, antiplasmodial, cytotoxicity

## Abstract

Four new flavones with modified prenyl groups, namely (*E*)-5-hydroxytephrostachin (**1**), purleptone (**2**), (*E*)-5-hydroxyanhydrotephrostachin (**3**), and terpurlepflavone (**4**), along with seven known compounds (**5**–**11**), were isolated from the CH_2_Cl_2_/MeOH (1:1) extract of the stem of *Tephrosia purpurea* subsp. *leptostachya*, a widely used medicinal plant. Their structures were elucidated on the basis of NMR spectroscopic and mass spectrometric evidence. Some of the isolated compounds showed antiplasmodial activity against the chloroquine-sensitive D6 strains of *Plasmodium falciparum,* with (*E*)-5-hydroxytephrostachin (**1**) being the most active, IC_50_ 1.7 ± 0.1 μM, with relatively low cytotoxicity, IC_50_ > 21 μM, against four cell-lines.

## 1. Introduction

*Tephrosia purpurea* (family Leguminosae) is one of the most widely distributed *Tephrosia* species and is found in tropical, subtropical, and other arid parts of the world. It consists of the four subspecies *purpurea*, *leptostachya*, *appolinea*, and *barbigera*, and four varieties, namely under subsp. *leptostachya* var. *leptostachya* and var. *pubescens*, and under subsp. *barbigera* var. *barbigera* and var. *rufescens* [1,2,3,4,5]. In Africa, a decoction of roots, leaves, and fruits of *Tephrosia purpurea* is given as a diuretic, for blood purification, and for the treatment of a cough and cold [6]. Its macerated leaves are used for curing diarrhoea and whooping cough in children [6]. In East Africa, its roots are used against stomach pains, while its leaves are used to treat snake bites and headaches. A decoction of its leaves and roots is used as a purgative [7], whereas that of the roots of *T. purpurea* subsp. *leptostachya* is employed for the treatment of schistosomiasis [6].

Phytochemical studies on *T. purpurea* collected from different parts of the world have resulted in the isolation of a wide variety of flavonoids; flavones [8,9], rotenoids [10], chalcones [11], and flavanones [12]. The crude extracts and pure compounds obtained from *T. purpurea* have shown a wide range of biological activities including antiplasmodial [12,13], anticancer [14], antacid [15], antidiabetic [16], analgesic and anti-inflammatory [17], and hepatoprotective [18] activities, and were also shown to be applicable to treat *Helicobacter pylori* infection [19]. Despite the presence of several subspecies and varieties of the taxa *T. purpurea*, the ethnobotanical, bioactivity, and phytochemical reports available so far have not been specific on the particular subspecies and variety. In order to better understand the relationship between *T. purpurea* and other species, the chemical variability among its subspecies and varieties has to be documented. With this in mind, the first phytochemical and biological report on *T. purpurea* subsp. *leptostachya* is reported here.

## 2. Results and Discussion

**Figure 1:**
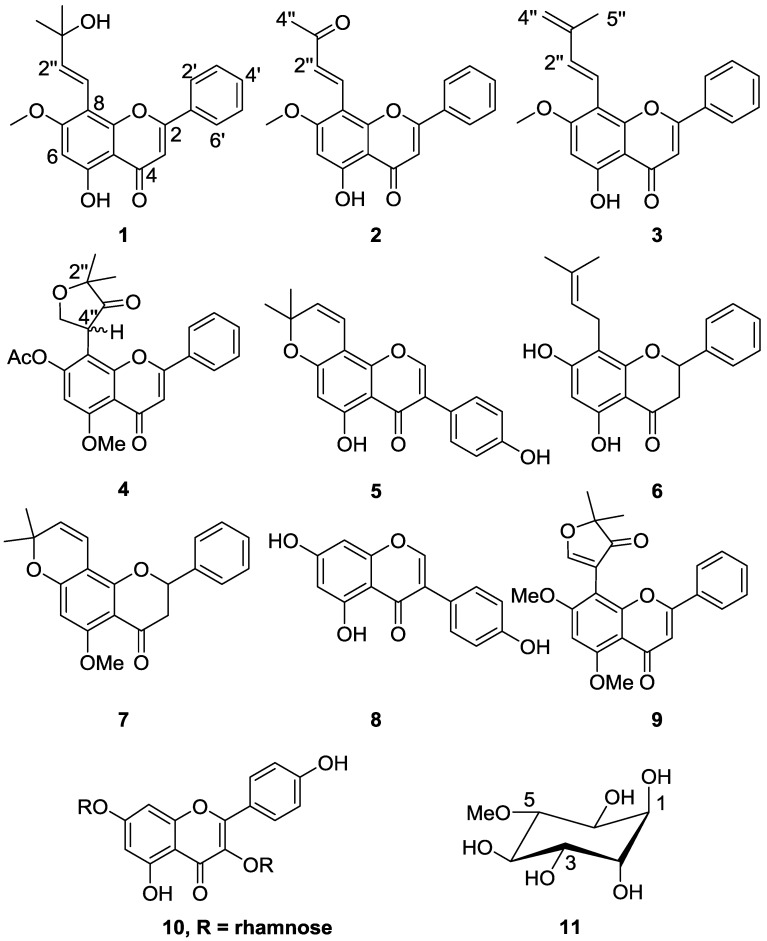
Structures of compounds isolated from *T. purpurea* subsp. *leptostachya.*

Extraction of the air dried stem of *T. purpurea* subsp. *leptostachya* with CH_2_Cl_2_/MeOH (1:1) at room temperature, followed by a combination of chromatographic separations, gave four new (**1**–**4**) and seven known (**5**–**11**) compounds (Figure 1).

Compound **1** was isolated as yellow crystals, and its molecular formula C_21_H_20_O_5_ was established from HRMS (*m*/*z* 352.1315) and ^1^H- and ^13^C-NMR data (Table 1, Figures S1–S6). The UV (λ_max_ 230, 270 and 310 nm), ^1^H (δ_H_ 6.67 for H-3), and ^13^C (δ_C_ 164.2 for C-2, 105.5 for C-3, and 182.9 for C-3) NMR spectral data suggested that this compound is a flavone derivative substituted with methoxy (δ_H_ 3.92; δ_C_ 56.1), hydrogen bonded hydroxyl (δ_H_ 13.08), and 2-methylbut-3-en-2-ol (Table 1, Tables S1–S6) substituents. The HMBC correlation of H-3 (δ_H_ 6.67) with C-2 (δ_C_ 164.2), C-4 (δ_C_ 182.9), and C-4a (δ_C_ 105.2) further supported the proposed flavone structure. Three sets of mutually coupled protons resonating at δ_H_ 7.91 (H-2′/6′), 7.52 (H-3′/5′), and 7.55 (H-4′) with corresponding carbons at δ_C_ 126.5 (C-2′/6′), 129.1 (C-3′/5′), and 131.9 (C-4′), respectively, were assigned to ring-B, which is unsubstituted (Table 1). The ^1^H-NMR data (Table 1) of **1** possesses a singlet at δ_H_ 6.40 (δ_C_ 95.3) on ring-A, which is hence trisubstituted with a methoxy (at C-7), a hydrogen bonded hydroxy (at C-5), and a (*E*)-2-methylbut-3-en-2-ol group. The HMBC correlations of the singlet at δ_H_ 6.40 with C-4a (δ_C_ 105.2), C-5 (δ_C_ 161.3), C-7 (δ_C_ 163.1), and C-8 (δ_C_ 105.3) allowed its assignment to H-6. Based on HMBC correlations, the methoxy group (δ_H_ 3.92, δ_C_ 56.1) was placed at C-7 (δ_C_ 163.1) and the hydrogen bonded hydroxy group (δ_H_ 13.08) at C-5, and the 2-methylbut-3-en-2-ol group could only be placed at C-8. This regiochemistry was confirmed by the HMBC correlation of OH-5 (δ_H_ 13.08) to C-4a (δ_C_ 105.2), C-5 (δ_C_ 161.3), and C-6 (δ_C_ 95.3)], and of the olefinic proton H-1″ (δ_H_ 6.85) to C-7 (δ_C_ 163.1) and C-8a (δ_C_ 154.1). The *J* = 16.5 Hz coupling between H-1″ (δ_H_ 6.85) and H-2″ (δ_H_ 6.70) is consistent with the *E*-configuration of the double bond of the 2-methylbut-3-en-2-ol group [20]. Therefore, compound **1** was characterized as (*E*)-5-hydroxy-8-(3-hydroxy-3-methylbut-1-en-1-yl)-7- methoxy-2-phenyl-4*H*-chromen-4-one. It is a 5-hydroxy derivative of *trans*-tephrostachin [20] and hence was given the trivial name (*E*)-5-hydroxytephrostachin.

The molecular formula of compound **2** was established as C_20_H_16_O_5_ from HRMS (*m*/*z* 336.0980), and ^1^H- and ^13^C-NMR data (Table 1, Figures S9–S13). Its UV spectrum (λ_max_ 230, 290, and 330 nm), along with its NMR spectra (Table 1), suggested that **2** had a flavone skeleton. Its ^1^H- and ^13^C-NMR spectra (Table 1) showed high similarities to those of **1**. Thus, ring-B of **2** is unsubstituted, while its ring-A is trisubstituted, with a hydroxy at C-5, a methoxy at C-7, and a modified prenyl group at C-8 (Table 1). The ^1^H-NMR spectral data further suggested the presence of *trans*-oriented and mutually coupled (*J* = 16.4 Hz) olefinic protons, which are deshielded (δ_H_ 8.06, H-1″, and δ_H_ 7.18, H-2″), suggesting a different substituent at C-8 of **2** as compared to **1**. Furthermore, a single, deshielded methyl signal (δ_H_ 2.41; δ_C_ 27.8) was observed, which along with an additional carbonyl signal (δ_C_ 199.1) showing HMBC correlations to H-1″ (δ_H_ 8.06) and H-2″ (δ_H_ 7.18), suggests that the C-8 substituent is the rare (*E*)-but-3-en-2-one group, similar to that reported for (2*S*)-5-hydroxy-7-methoxy-8-[(*E*)-3-oxo-1-butenyl]flavanone [21], and for erylivingstone F [22]. Based on the above spectroscopic data, compound **2** was characterized as (*E*)-5-hydroxy-7-methoxy-8-(3-oxobut-1-en-1-yl)-2-phenyl-4*H*-chromen-4-one and was given the trivial name purleptone.

Compound **3** ([M + 1]^+^ *m*/*z* 335.1227, C_21_H_18_O_4_) was also found to be a flavone derivative (λ_max_ 230, 280 and 310 nm), whose ^1^H- and ^13^C-NMR spectra (Table 1, Figures S16–S21) showed close similarities to those of **1** and **2**. It was found to have an unsubstituted ring-B, and trisubstituted ring-A with hydroxy at C-5, methoxy at C-7, and a modified prenyl group at C-8. The structure of the latter substituent was established to be (*E*)-3-methylbuta-1,3-dien-1-yl from the ^1^H- and ^13^C-NMR spectral data (Table 1), and was confirmed by the HMBC correlations of CH_2_-4″ (δ_H_ 5.10) with C-2″ (δ_C_ 135.4), C-3″ (δ_C_ 142.9), and C-5″ (δ_C_ 18.2). The placement of this group at C-8 was established from the HMBC correlations of H-2″ (δ_H_ 6.29) to C-8 (δ_C_ 106.0), C-3″ (δ_C_ 142.9), C-4″ (δ_C_ 116.8), and C-5″ (δ_C_ 18.2), and of H-5″ (δ_H_ 2.06) with C-2″ (δ_C_ 135.4), C-3″ (δ_C_ 142.9), and C-4″ (δ_C_ 116.8). In agreement with this, H-1″ also showed HMBC correlation with C-7 (δ_C_ 163.2), C-8a (δ_C_ 154.2), C-2″ (δ_C_ 135.4), and C-3″ (δ_C_ 142.9). Compound **3** was therefore characterized as (*E*)-5-hydroxy-7-methoxy-8-(3- methylbuta-1,3-dien-1-yl)-2-phenyl-4*H*-chromen-4-one, and was given the trivial name (*E*)-5-hydroxyanhydrotephrostachin as it is structurally closely related to anhydrotephrostachin [20].

The structure of compound **4** ([M + 1]^+^, *m*/*z* 423.1465, C_24_H_22_O_7_), also a flavone, was established from ^1^H- and ^13^C-NMR data (Table 2, Figures S24–S29), as well as from its UV spectrum (λ_max_ 230, 260, and 310 nm). Its NMR spectra (Table 2) revealed the presence of an unsubstituted ring-B (δ_H_ 7.70, δ_C_ 126.3 (H-2′/6′), δ_H_ 7.45_,_ δ_C_ 128.7 (H-3′/5′), and δ_H_ 7.49, δ_C_ 131.1 (H-4′ *m*)), a methoxy (δ_H_ 3.96, δ_C_ 56.7) at C-5, an acetate [(δ_H_ 2.11, δ_C_ 21.4 (Me), δ_C_ 170.0 (C=O)] at C-7, and a modified prenyl group in the form of a tetrahydrofuran ring at C-8 (Table 2), similar to terpurinflavone [12] and tephroglabrin [23]. The presence of an additional carbonyl (δ_C_ 206.1) and two geminal methyl groups (δ_H_ 1.57, δ_C_ 24.0 and δ_H_ 1.65, δ_C_ 23.9), and three mutually coupled protons at δ_H_ 4.95 (*dd, J* = 6.1, 10.2 Hz), δ_H_ 4.90 (*dd*, *J* = 6.1, 8.8) and δ_H_ 4.84 (*dd*, *J* = 6.1, 8.8 Hz) indicated that the C-8 substituent was a 5,5-dimethyl-4-oxo-tetrahydrofuran-3-yl group. In agreement with this, H-4″ (δ_H_ 4.95), H-5″ (δ_H_ 4.90), and 2″-(Me)_2_ (δ_H_ 1.57 and 1.65) showed HMBC correlations to the carbonyl carbon C-3″ (δ_C_ 206.1). The HMBC correlation of H-4″ (δ_H_ 4.95) with C-7; H-6 (δ_H_ 6.41) with C-4a (δ_C_ 109.1), C-5 (δ_C_ 162.9), C-7 (δ_C_ 166.3), and C-8 (δ_C_ 103.9); and the OMe (δ_H_ 3.96) with C-5 (δ_C_ 162.9) confirmed the substitution pattern of this ring. The coupling constant *J* = 10.2 Hz of H-4″ and H-5″ indicated a 1,2-diaxial orientation of these protons [12]. Hence, compound **4** was characterized as 8-(5,5-dimethyl-4-oxotetrahydrofuran-3-yl)-5-methoxy-4-oxo-2-phenyl-4H-chromen-7-yl acetate and was given the trivial name terpurlepflavone.

The known compounds were identified as derrone (**5**) [24], glabranin (**6**) [25], obovatin methyl ether (**7**) [26], genistein (**8**) [27], tachrosin (**9**) [28], kaempferitrin (**10**) [29], and d-pinitol (**11**) [30] by a comparison of their spectroscopic data (Tables S1 to S7) with that available in the literature. The major flavones of this plant were tested for antiplasmodial activity against the D6 strain of *Plasmodium falciparum* (Table 3). Among these, (*E*)-5-hydroxytephrostachin (**1**) showed good activity, IC_50_ 1.7 μM), while terpurlepflavone (**4**) and tachrosin (**9**) showed low antiplasmodial activities. The compounds were also tested for cytotoxicity against two non-tumoral and two cancerous cell-lines (Table 3). Most of these did not show cytotoxicity (IC_50_ > 100 μM), while compound **1** showed IC_50_ between 21–100 μM, which is still significatly lower than its antiplasmodial activity with a selectivity index > 12. The results observed here demonstrate the potential of flavones as antiplasmodial agents, parallel to the in vitro and in vivo antiplasmodial activites reported earlier for some flavones [12,31].

## 3. Materials and Methods

### 3.1. General Experimental Procedure

UV spectra were recorded on a Specord S600 (Analytik Jena AG, Jena, Germany) spectrophotometer. Melting points were obtained on a Büchi Melting point B-545 (Flawil, Switzerland) apparatus, and optical rotations were measured on Perkin Elmer 341-LC (Perkin Elmer, Wellesley, MA, USA), whereas CD experiments were run on a Jasco J-715 spectropolarimeter (Jasco, Corp., Tokyo, Japan). NMR spectra were acquired on a Bruker Avance III HD 800 spectrometer (Bruker BioSpin AG, Fallanden, Switzerland) equipped with a TXO cryogenic probe using the residual solvent peak as the reference. Analytical reversed phase liquid chromatography (RP-HPLC)—mass spectrometry (MS) was performed on a API SCIEX 150 EX Perkin Elmer (Perkin Elmer, Waltham, MA, USA) ESI-MS (30 eV) connected to a Perkin Elmer gradient pump system and a C8 column (120 Å, 4 μm, 4.6 mm × 50 mm) using gradients of acetonitrile/water (CH_3_CN/H_2_O) with 1% formic acid (HCOOH) as the mobile phase at a flow rate of 1 mL/min. TLC was carried out on Merck pre-coated silica gel 60 F254 plates (Merck, Darmstadt, Germany). Column chromatography was run on silica gel 60 (70–230 mesh). Gel filtration was done on Sephadex LH-20 (Fluka, Buchs, Switzerland). Preparative HPLC was carried out on a Waters 600E instrument using the Chromulan (Pikron Ltd., Praha, Czech Republic) software and a RP-C_8_ Kromasil® (250 mm × 55 mm, Kromasil, Bohus, Sweden) column with an H_2_O/MeOH solvent system for elution. HRESIMS were obtained with a Q-TOF-LC/MS spectrometer (Stenhagen Analyslab AB, Gothenburg, Sweden) using a 2.1 mm × 30 mm, 1.7 μm RPC18 column and a H_2_O–CH_3_CN gradient system (5:95–95:5 gradient and 0.2% formic acid).

### 3.2. Plant Material

The stems of *Tephrosia purpurea* subsp. *leptostachya* were collected in April 2015 from the Kilungu hills in Makueni County, Kenya. The plant specimen was identified by Mr. Patrick C. Mutiso of the Herbarium, School of Biological Sciences, University of Nairobi, where a voucher specimen (Mutiso-841/April 2015) was deposited.

### 3.3. Extraction and Isolation

The air dried and ground stems (2 kg) of *T*. *purpurea* subsp. *leptostachya* were extracted with CH_2_Cl_2_/MeOH (1:1) for seven days at 20–25 °C by percolation (3 × 2 L) to yield a dark yellow paste (80 g, 4%). Hence, it was soaked for 24 h with 2 L solvent, filtered, and concentrated using a rotatory evaporator. This procedure was then repeated three times. A portion of the extract (31 g) was subjected to column chromatography over silica gel (300 g) eluting with *iso*-hexane containing increasing amounts of EtOAc. The fraction that eluted with 3% EtOAc in *iso*-hexane was purified by gel filtration on Sephadex LH-20 (eluent: CH_2_Cl_2_/MeOH; 1:1) to give **2** (16.2 mg, ≥97% purity) and **3** (23.4 mg, ≥97% purity). The eluent with 5% EtOAc in *iso*-hexane was first separated over Sephadex LH-20 (CH_2_Cl_2_/MeOH; 1:1) followed by preparative HPLC (20:80 MeOH/H_2_O–100% MeOH gradient elution for 20 min with flow rate 8 mL/min) to give **5** (derrone, 28 mg, ≥98% purity) [24], **6** (glabranin, 52 mg, ≥98% purity) [25], **7** (obovatin methyl ether, 47 mg, ≥99% purity) [26], and **8** (genistein, 53 mg, ≥98% purity) [27]. Elution with 6% EtOAc in *iso*-hexane gave a yellow solid which was recrystallized from CH_2_Cl_2_/MeOH (1:1) to give **1** (550 mg, ≥99% purity). Further elution with 8% EtOAc in *iso*-hexane gave **4** (67.5 mg, ≥99% purity); the eluent with 9% EtOAc in *iso*-hexane gave **9** (tachrosin, 158 mg, >99% purity) [28]; and the 10% EtOAc in *iso*-hexane eluent gave **10** (kaempferitrin, 97 mg, >99% purity) [29]. Fraction elution with 15% EtOAc in *iso*-hexane gave **11** (d-pinitol, 650 mg, >99% purity) [30].

(*E*)*-5-Hydroxytephrostachin* (**1**)*:* Yellow crystals (CH_2_Cl_2_/MeOH; 1:1). mpt 160–162 °C. UV λ_max_ (CH_2_Cl_2_): 230, 270 and 310 nm. ^1^H- and ^13^C-NMR (Table 1). EIMS *m*/*z* (rel. int.) 353.6 [M]^+^ (100). HRMS [M]^+^ *m*/*z* 352.1315 C_21_H_20_O_5_ (Calculated: 352.1311).

*Purleptone* (**2**)*:* Colourless amorphous solid. UV λ_max_ (CH_2_Cl_2_): 230, 290 and 330 nm.^1^H- and ^13^C-NMR (Table 1). EIMS *m*/*z* (rel. int.) 337 [M]^+^ (100). HRMS [M]^+^ *m*/*z* 336.0980 C_20_H_16_O_5_ (Calculated: 336.0998).

(*E*)*-5-Hydroxyanhydrotephrostachin* (**3**)*:* Colourless amorphous solid. UV λ_max_ (CH_2_Cl_2_): 230, 280 and 310 nm. ^1^H- and ^13^C-NMR (Table 1). EIMS *m*/*z* (rel. int.) 336.1276 [M]^+^. HRMS [M + 1]^+^ *m*/*z* 335.1227 C_21_H_18_O_4_ (Calculated: 335.1283).

*Terpurlepflavone* (**4**)*:* White amorphous solid. m.pt 210–214 °C. UV λ_max_(CH_2_Cl_2_): 230, 260 and 310 nm. CD (MeOH) λ nm (Δε; M^−1^·cm^−1^): (122.83)_221_; (−58.17)_212._[α]_D_^20^ +14.00° (c 0.001, MeOH). ^1^H- and ^13^C-NMR (Table 2). EIMS *m*/*z* (rel. int.) 423 [M]^+^. HRMS [M + 1]^+^ *m*/*z* 423.1465 C_24_H_22_O_7_ (Calculated: 423.1444).

### 3.4. In Vitro Antiplasmodial Activity

The pure compounds were assayed using a non-radioactive assay technique as described by Smilkstein et al., 2004 [32] with modifications given in the literature [12,33].

### 3.5. Cell Culture

A549, HepG2, and non-tumoral cells were all purchased from ATCC. Cells were cultured in RPMI 1640 medium supplemented with 10% fetal bovine serum and antibiotics penicillin (50 U/mL) and streptomycin (50 μg/mL; Invitrogen, Paisley, Scotland, UK). All cell cultures were incubated at 37 °C in a 5% humidified CO_2_ incubator.

### 3.6. Cytotoxicity Assay

All tested compounds were dissolved in DMSO at a final concentration of 50 mmol/L and stored at −20 °C before use. Cytotoxicity was assessed by using the 3-(4,5-dimethylthiazol-2-yl)-2,5-diphenyltetrazolium bromide (MTT) (5.0 mg/mL) assay as previously described [34]. Briefly, 4 × 10^3^ cells per well were seeded in 96-well plates before drug treatments. After overnight culture, the cells were then exposed to different concentrations of selected compounds (0.039–100 μmol/L) for 72 h. Cells without drug treatment were used as the control. Subsequently, MTT (10 μL) solution was added to each well and incubated at 37 °C for 4 h followed by the addition of 100 μL solubilization buffer (10% SDS in 0.01 mol/L HCl) and overnight incubation. A_570_ nm was then determined in each well on the next day. The percentage of cell viability was calculated using the following formula: Cell viability (%) = A_treated_/A_control_ × 100. Data were obtained from three independent experiments and the standard error was calculated.

## 4. Conclusions

Four new prenylflavones with seven known compounds were isolated from the stem of *Tephrosia purpurea* subsp. *leptostachya.* The isolated flavones were tested for antiplasmodial activity against the D6 strain of *Plasmodium falciparum*. Among these, (*E*)-5-hydroxytephrostachin (**1**) showed good activity (IC_50_ 1.7 μM). The compounds were also tested for cytotoxicity against two non-tumoral and two cancerous cell-lines. Most of these did not show cytotoxicity (IC_50_ > 100 μM), while compound **1** showed IC_50_ between 21–100 μM.

## Figures and Tables

**Table 1:** ^1^H- (800 MHz) and ^13^C- (200 MHz) NMR data for compounds **1**, **2**, and **3** (in CDCl_3_) at 25 °C.

Position	1			2			3		
	δ_C_ (ppm)	δ_H_, *m* (*J* in Hz)	HMBC (H→C)	δ_C_	δ_H_, *m* (*J* in Hz)	HMBC (H→C)	δ_C_	δ_H_, *m* (*J* in Hz)	HMBC (H→C)
2	164.2			164.6			164.2		
3	105.5	6.57 *s*	C-2, C-4, C-4a, C-1′	106.2	6.74 *s*	C-2, C-4, C-4a, C-1′	105.5	6.71 *s*	C-2, C-4, C-4a, C-1′
4	182.9			182.6			183.0		
4a	105.2			105.4			105.3		
5	161.3			164.2			161.4		
5-OH		13.08 *s*	C-4a, C-5, C-6		13.41 *s*	C-4a, C-5, C-6		13.11 *s*	C-4a, C-5, C-6
6	95.3	6.40 *s*	C-4a, C-5, C-7, C-8	95.6	6.40 *s*	C-4a, C-5, C-7, C-8	95.4	6.45 *s*	C-4a, C-5, C-7, C-8
7	163.1			165.0			163.2		
8	105.3			103.4			106.0		
8a	154.1			156.0			154.2		
1′	131.5			131.5			131.5		
2′,6′	126.5	7.91 *m*	C-2, C-4′, C-2′, C-6′	126.5	7.92 *m*	C-2, C-4′, C-2′, C-6′	126.4	7.93 *m*	C-2, C-4′, C-2′, C-6′
3′,5′	129.1	7.52 *m*	C-1′, C-3′, C-5′	129.4	7.59 *m*	C-1′, C-3′, C-5′	129.2	7.54 *m*	C-1′, C-3′, C-5′
4′	131.9	7.55 *m*	C-2′, C-6′	132.2	7.59 *m*	C-2′, C-6′	132.0	7.56 *m*	C-2′, C-6′
1″	114.9	6.85, *d* (16.5)	C-7, C-8a, C-2″, C-3″	132.0	8.06, *d* (16.4)	C-7, C-8a, C-2″, C-3″	117.5	6.83, *d* (16.5)	C-7, C-8a, C-2″, C-3″
2″	141.3	6.70, *d* (16.5)	C-8, C-3″, 3″-Me_2_	128.8	7.18, *d* (16.4)	C-8, C-3″, C-4″	135.4	6.29, *d* (16.5)	C-8, C-3″, C-4″, C-5″
3″	71.5			199.1			142.9		
3″-Me_2_	30.0	1.50 *s*	C-2″, C-3″, 3″-Me_2_						
4″				27.8	2.41 *s*	C-2″, C-3″	116.8	5.10 *s*	C-2″, C-3″, C-5″
5″							18.2	2.06 *s*	C-2″, C-3″, C-4″
7(OMe)	56.1	3.92 *s*	C-7	56.4	4.01 *s*	C-7	56.2	3.97 *s*	C-7

**Table 2:** ^1^H- (800 MHz) and ^13^C- (200 MHz) spectroscopic data for compound **4** (CDCl_3_) at 25 °C.

Position	δ_C_	δ_H_, *m* (*J* in Hz)	HMBC (H→C)
2	160.6		
3	110.1	6.55 *s*	C-2, C-4, C-4a, C-1′
4	177.2		
4a	109.1		
5	162.9		
6	91.1	6.41 *s*	C-4a, C-5, C-7, C-8
7	166.3		
8	103.9		
8a	154.9		
1′	131.7		
2′,6′	126.3	7.70 *m*	C-2, C-4′, C-2′, C-6′
3′,5′	128.7	7.45 *m*	C-1′, C-3′, C-5′
4′	131.1	7.49 *m*	C-2′, C-6′
2″	83.9		
3″	206.1		
4″	47.7	4.95 *dd* (10.2, 6.1)	C-7, C-8, C-8a, C-2″, C-3″, C-5″
5″	75.8	4.90 *dd* (10.2, 8.8)	C-7, C-8, C-3″, C-4″
		4.84 *dd* (6.1, 8.8)	C-7, C-8, C-3″, C-4″
2″-Me	24.0	1.57 *s*	C-2″, C-3″, 2″-Me
2″-Me	23.9	1.65 *s*	C-2″, C-3″, 2″-Me
5-OMe	56.7	3.96 *s*	C-5
7-COMe	170.0		
7-COMe	21.4	2.11 *s*	7-COMe

**Table 3:** In vitro antiplasmodial activity and cytototoxicity of compounds **1**, **2**, **4** and **9** (IC_50_, μM).

Samples	Antiplasmodial Activity against *P. falciparum*	Cytotoxicity			
	D6	LO2 *	BEAS *	A549 **	HepG2 **
(*E*)-5-Hydroxytephrostachin (**1**)	1.7 ± 0.1	21.7 ± 4.8	24.5 ± 2.7	76.1 ± 2.9	>100
Purleptone (**2**)	NT	>100	>100	>100	>100
Terpurlepflavone (**4**)	14.8 ± 3.2	>100	>100	>100	>100
Tachrosin (**9**)	27.1 ± 3.2	>100	>100	>100	>100
Chloroquine	0.037 ± 0.003				
Artesunate-Mefloquine	0.075 ± 0.006				

* Non-tumoral cell: LO2, Immortal human hepatic cell line; BEAS, Lung/bronchus cell line (epithelial virus transformed); ** Cancer cell: A549, adenocarcinomic human alveolar basal epithelial cells; HepG2, human liver cancer cell line; NT = Not Tested.

## Supplementary Materials

The Supplementary Materials are available online. NMR, UV and MS spectra for all new compounds and spectral data for the known compounds are available as Supporting Information.
