# Supplementary material for: Four Prenylflavone Derivatives with Antiplasmodial Activities from the Stem of Tephrosia purpurea subsp. leptostachya
Source: Molecules. 2017 Sep 10;22(9):1514. doi: 10.3390/molecules22091514 (PMC6151588; doi:10.3390/molecules22091514)
Supplement: Supplementary file 1 [file molecules-22-01514-s001.pdf]

Four prenylflavone derivatives with antiplasmodial activities from the stems of *Tephrosia purpurea* subsp. *leptostachya*

Yoseph Atilaw, Lois Muiva-Mutisya, Albert Ndakala, Hoseah M. Akala, Redemptah Yeda, Yu J. Wu, Paolo Coghi, Vincent K.W. Wong, Máté Erdélyi, Abiy Yenesew

Table of Contents

Figure	Content Page
Figs. S1 –S6 ^{1}H & ^{13}C NMR; COSY; NOESY; HSQC & HMBC correlations of compound **1**	3-8
Fig. S7 HRMS of compound **1**	9
Fig. S8 UV spectrum of compound **1**	10
Figs. S9 –S13 ^{1}H & ^{13}C NMR; NOESY; HSQC & HMBC correlations of compound **2**	11-15
Fig. S14 HRMS of compound **2**	16
Fig. S15 UV spectrum of compound **2**	17
Fig. S16-S21 1H; 13C; COSY; NOESY; HSQC & HMBC correlations of compound **3**	18-23
Fig. S22 HRMS of compound **3**	24
Fig. S23 UV spectrum of compound **3**	25
Fig. S24-S29 ^{1}H and ^{13}C; COSY; NOESY; HSQC & HMBC correlations of compound **4**	26-31
Fig. S30 HRMS of compound **4**	32
Fig. S31 UV spectrum of compound **4**	33
Table S11H (800 MHz) and ^{13}C (200 MHz) NMR spectroscopic data for compound **5**, CDCl3	34
Table S21H (800 MHz) and ^{13}C (200 MHz) NMR spectroscopic data for compound **6**, CDCl3	35
Table S31H (800 MHz) and ^{13}C (200 MHz) NMR spectroscopic data for compound **7**, CDCl3	36
Table S41H (800 MHz) and ^{13}C (200 MHz) NMR spectroscopic data for compound **8**, CDCl3	37
Table S51H (800 MHz) and ^{13}C (200 MHz) NMR spectroscopic data for compound **9**, CDCl3	38
Table S61H (800 MHz) and ^{13}C (200 MHz) NMR spectroscopic data for compound **10**, CDCl3	39
Table S71H (800 MHz) and ^{13}C (200 MHz) NMR spectroscopic data for compound **11**, CDCl3	40

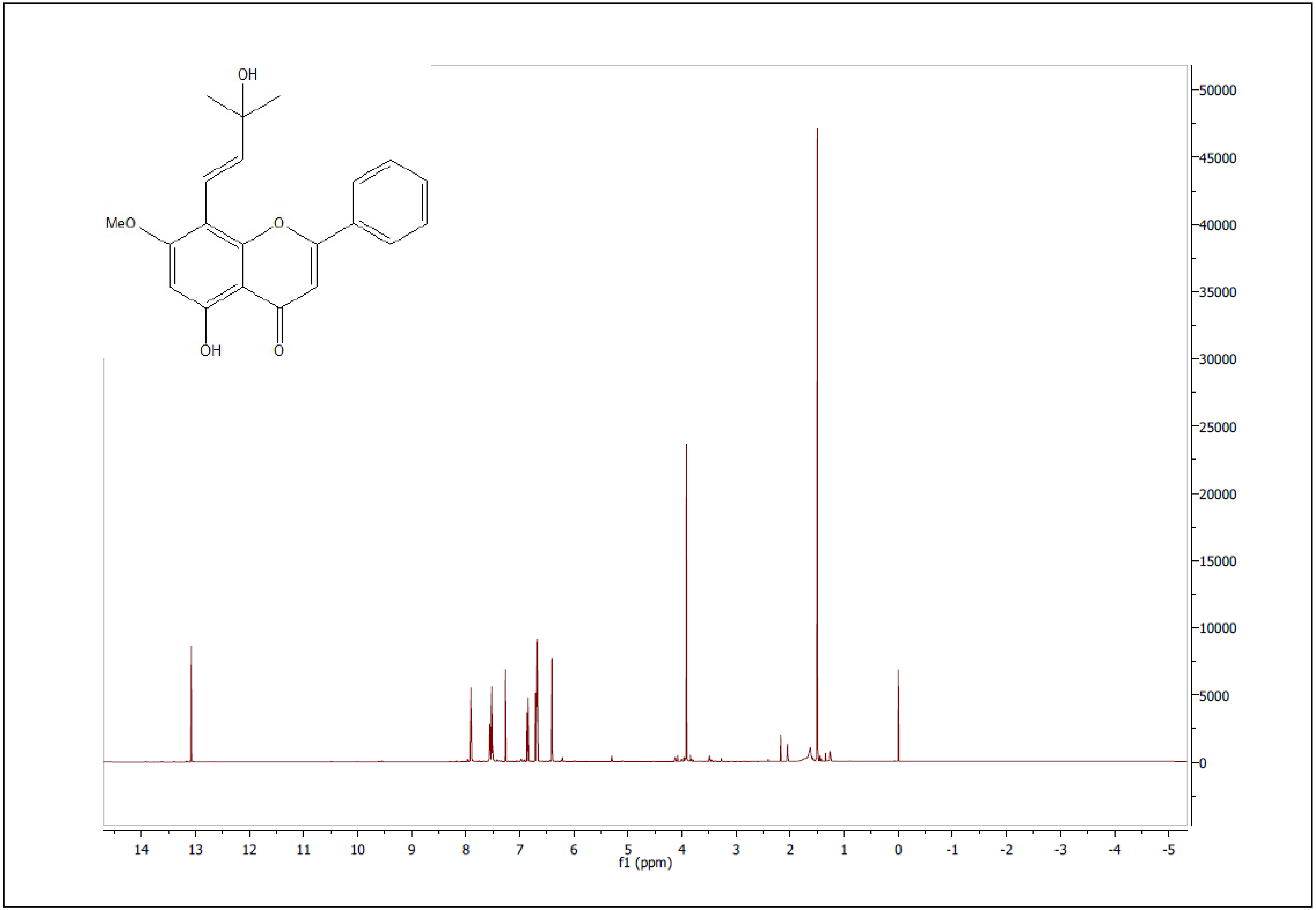

Fig. S1: ^{1}H NMR spectrum of compound 1 (800 MHz; $CDCl_3$)

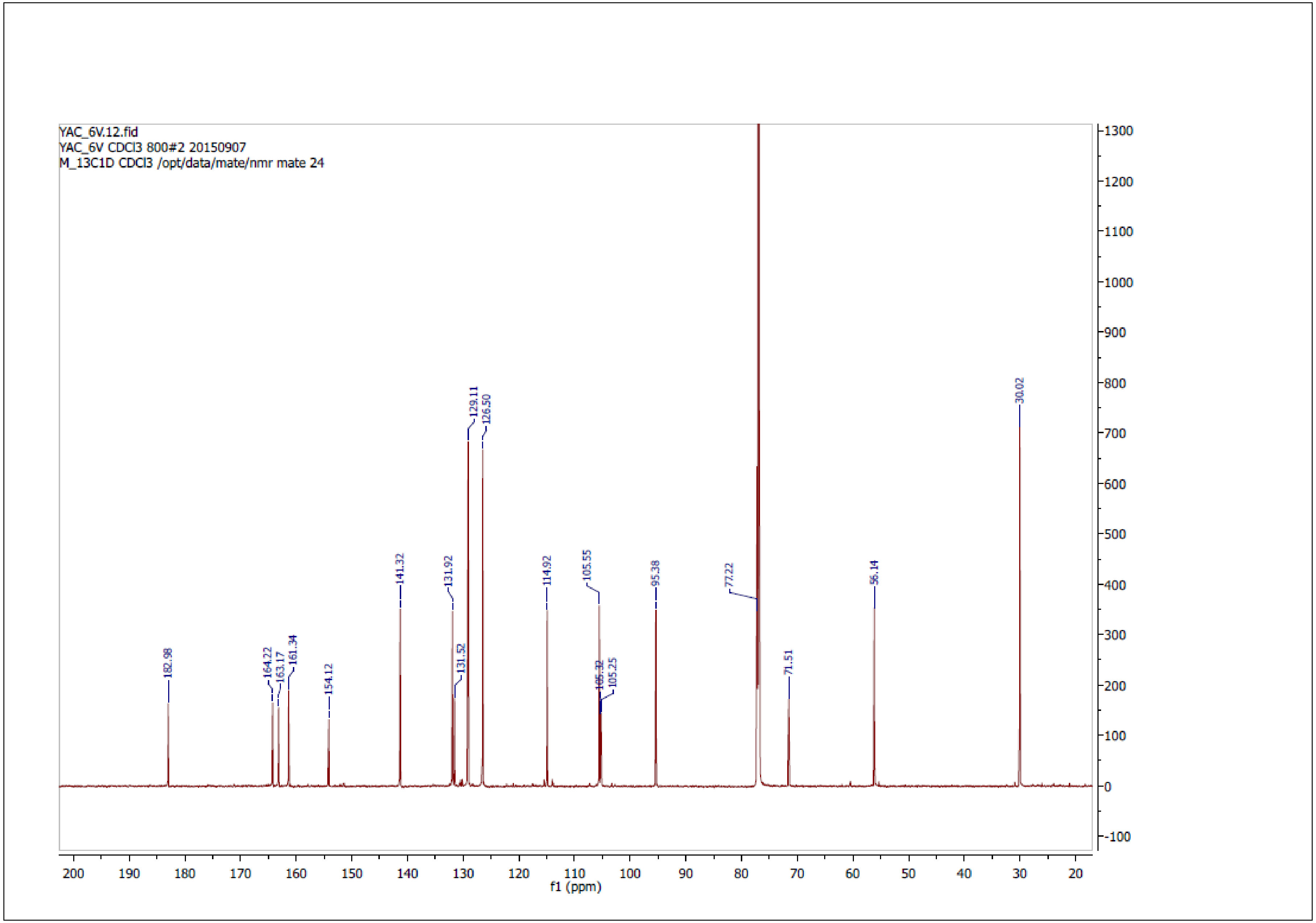

Fig. S2: ^{13}C NMR spectrum of compound 1 (200 MHz; $CDCl_3$)

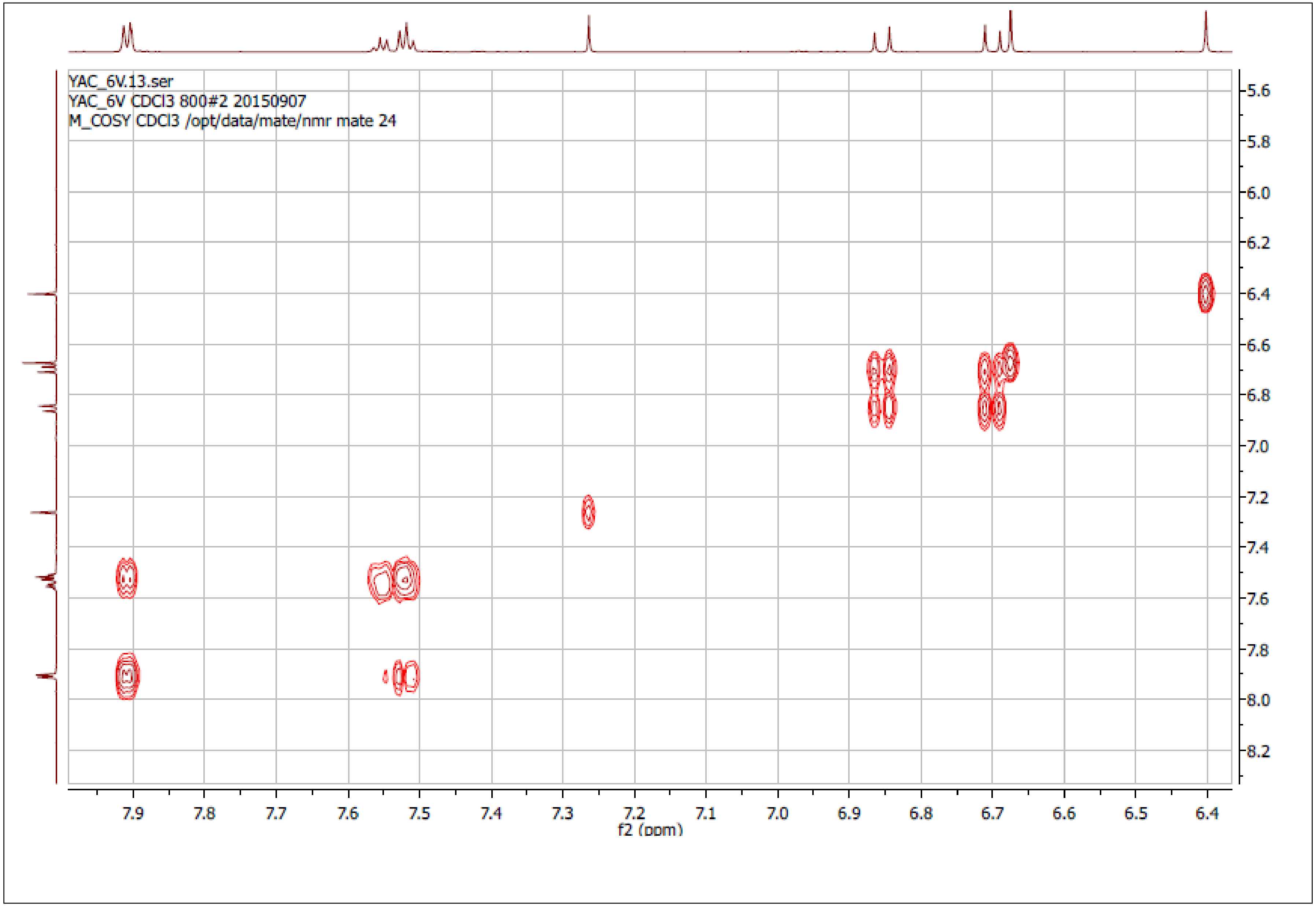

Fig. S3: COSY spectrum of compound **1** ($CDCl_3$)

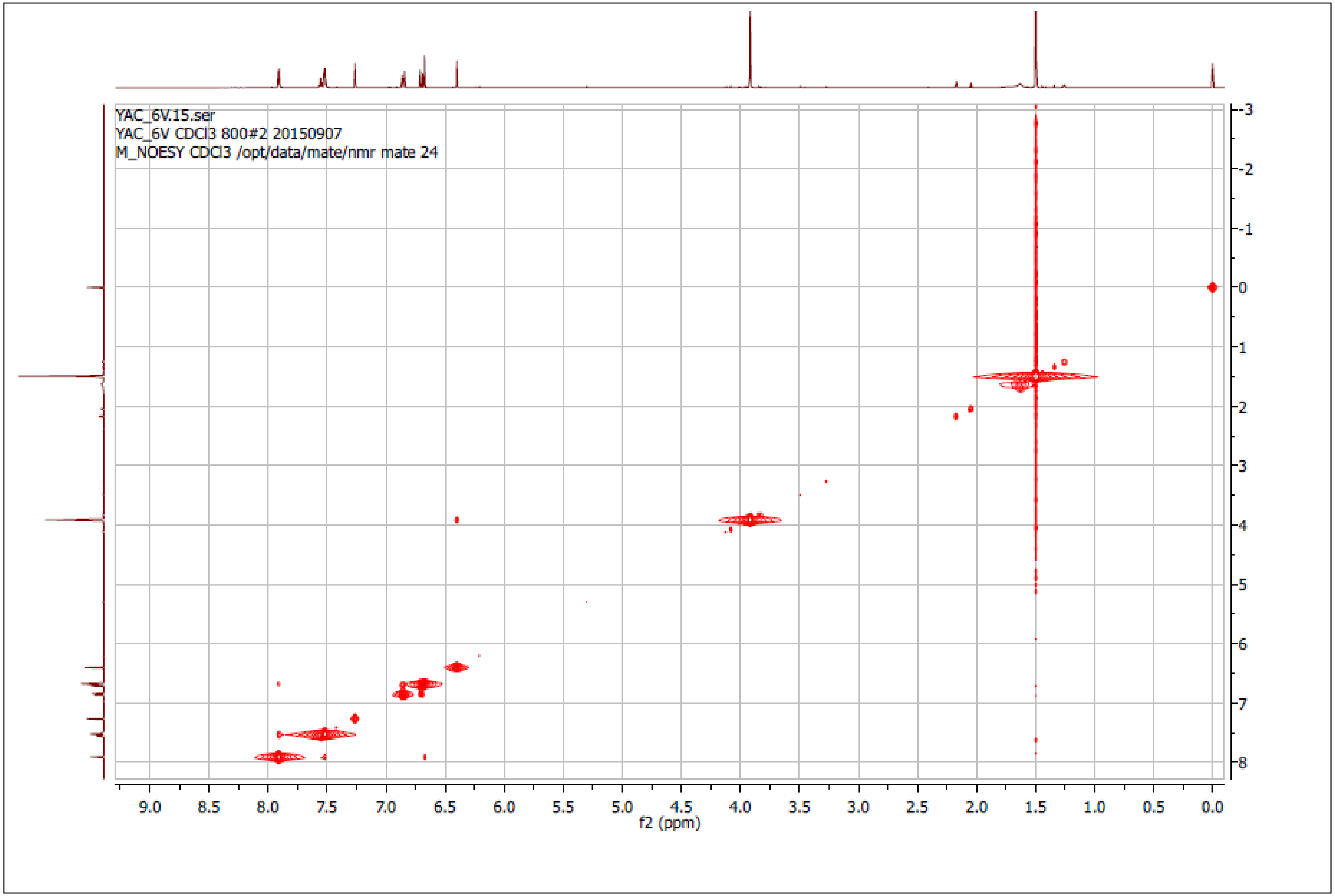

Fig. S4: NOESY spectrum of compound **1** ($CDCl_3$)

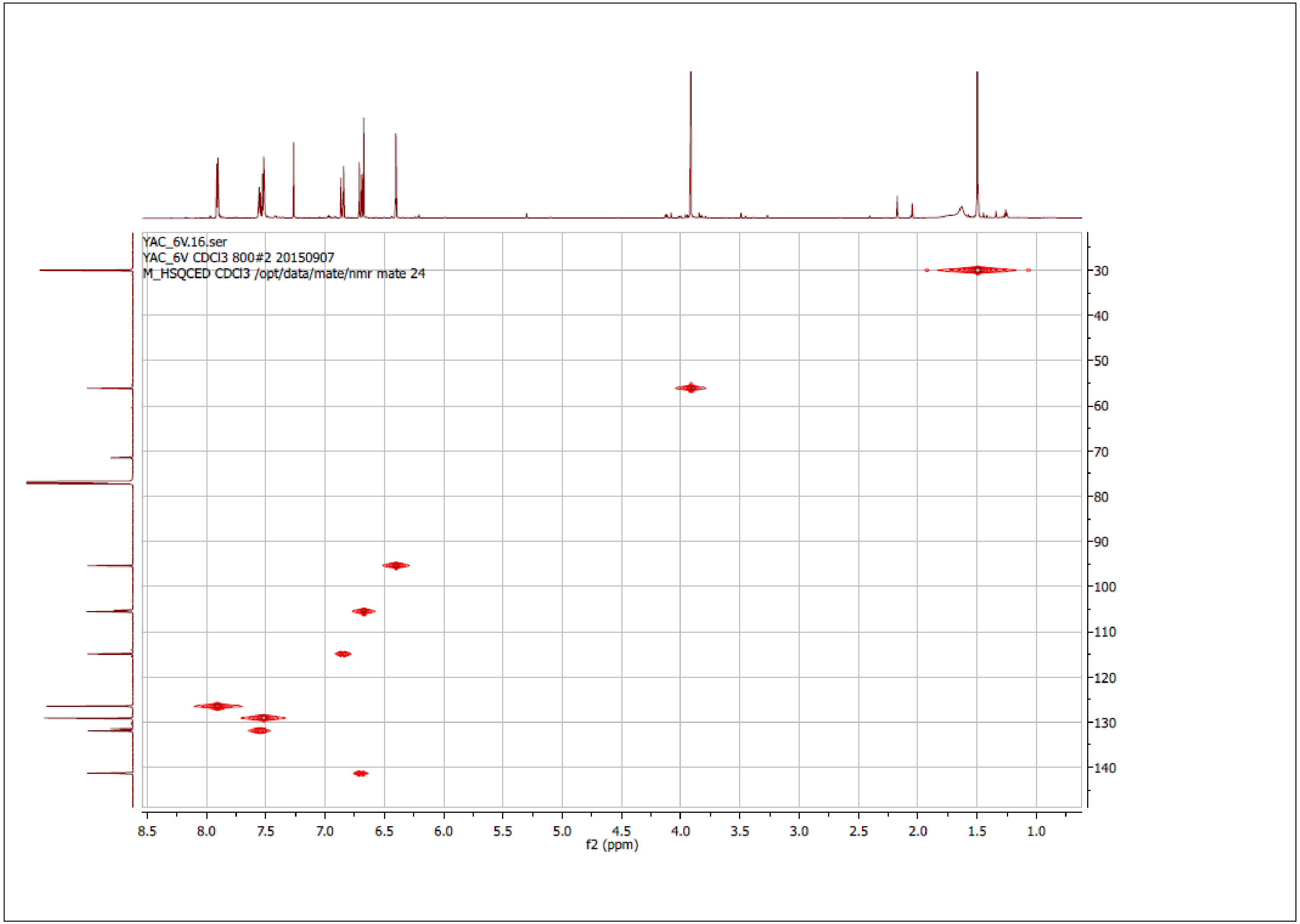

Fig. S5: HSQC spectrum of compound **1** ($CDCl_3$)

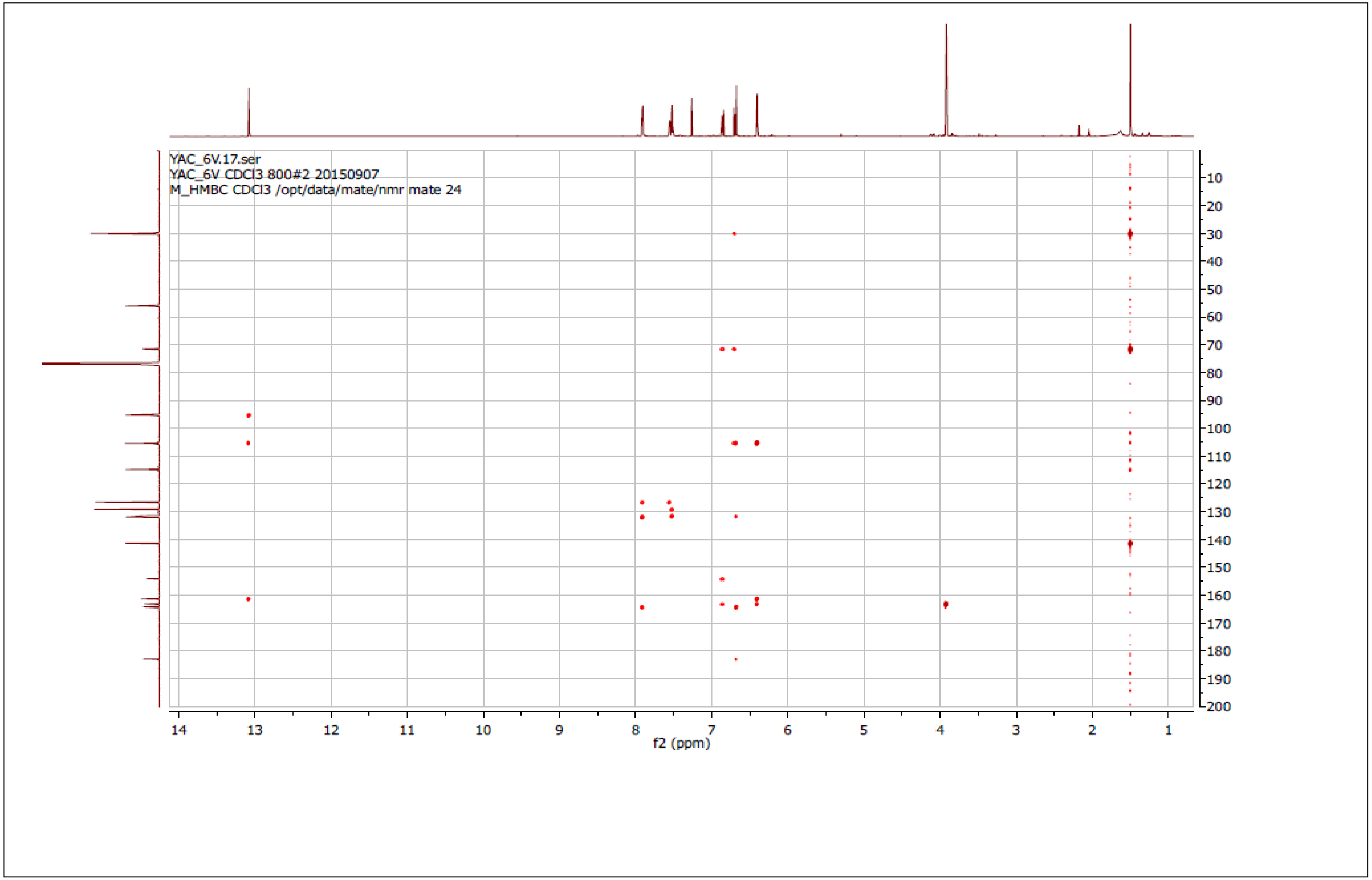

Fig. S6: HMBC spectrum of compound **1** ($CDCl_3$)

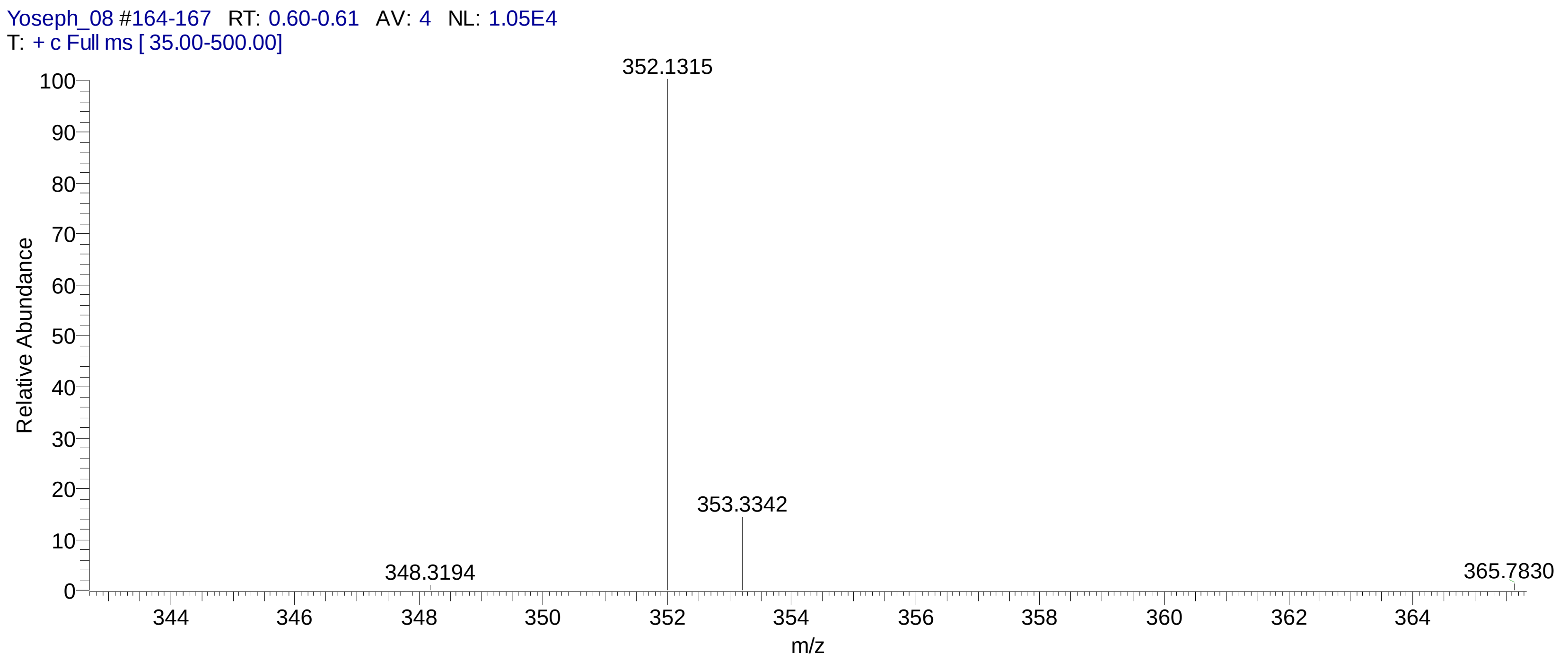

Fig. S7: HRMS of compound **1**

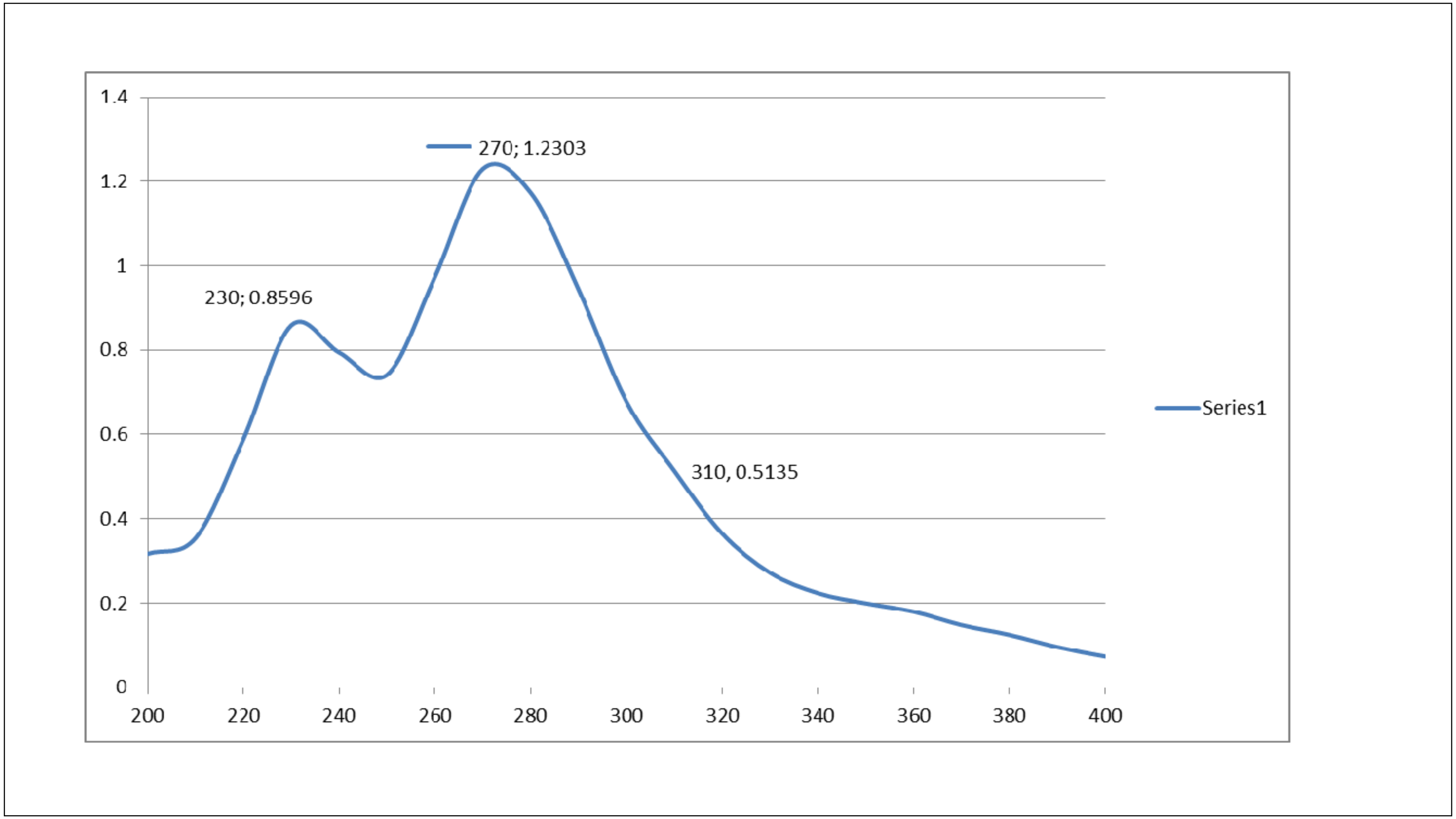

Fig. S8: UV spectrum of compound **1**

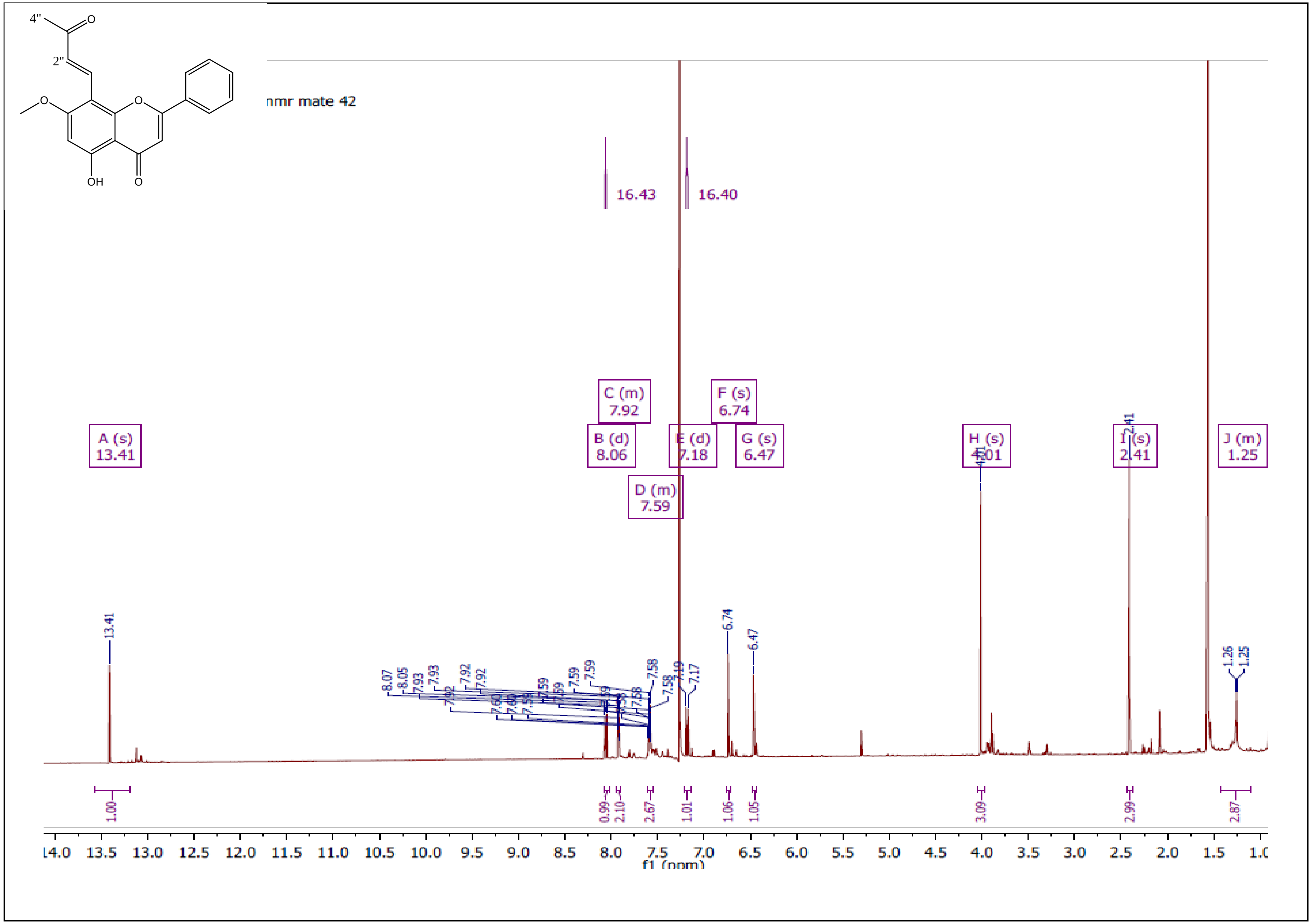

Fig. S9: ^{1}H NMR spectrum of compound **2** (800 MHz; $CDCl_3$)

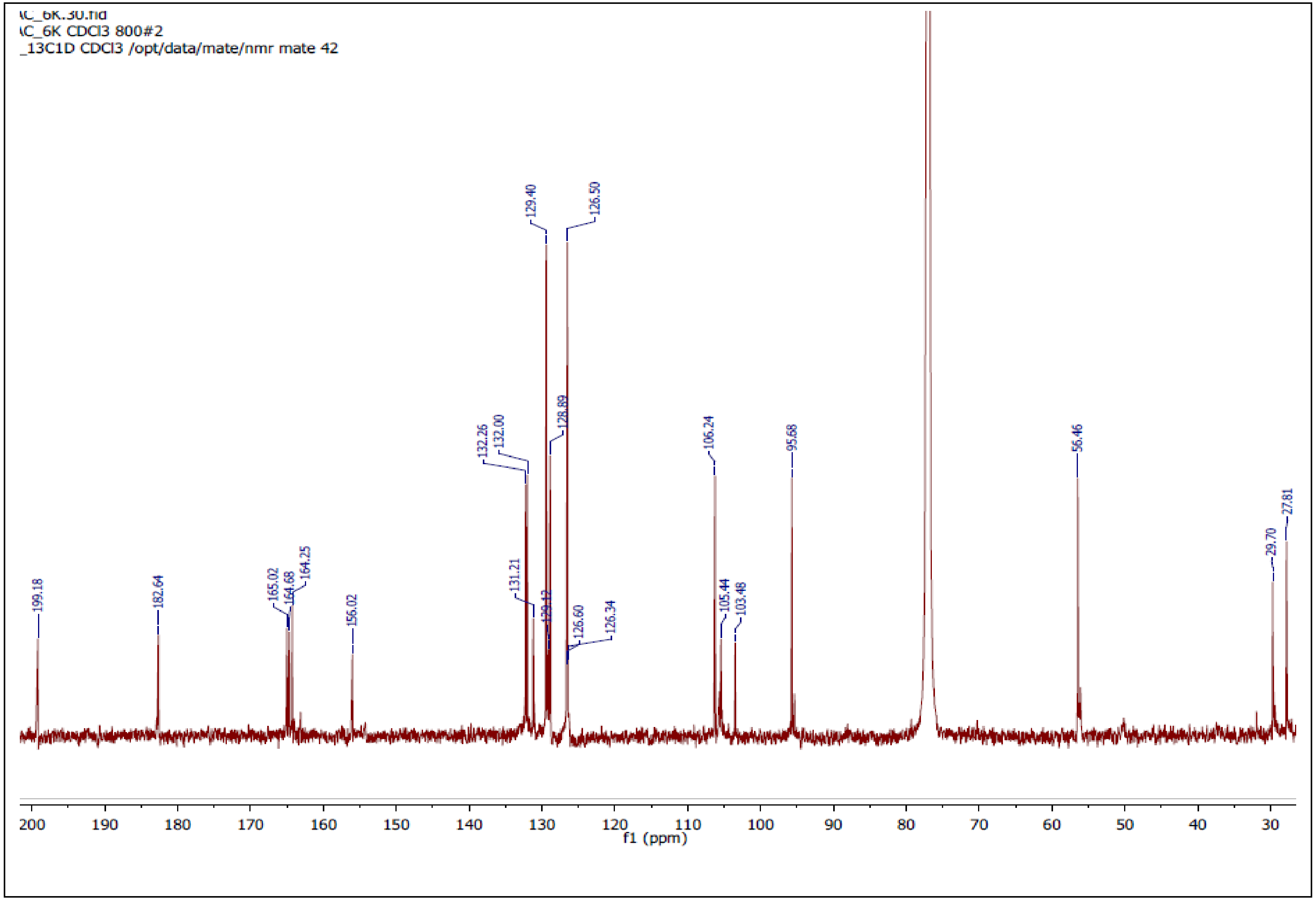

Fig. S10: ^{13}C NMR spectrum of compound **2** (200 MHz; $CDCl_3$)

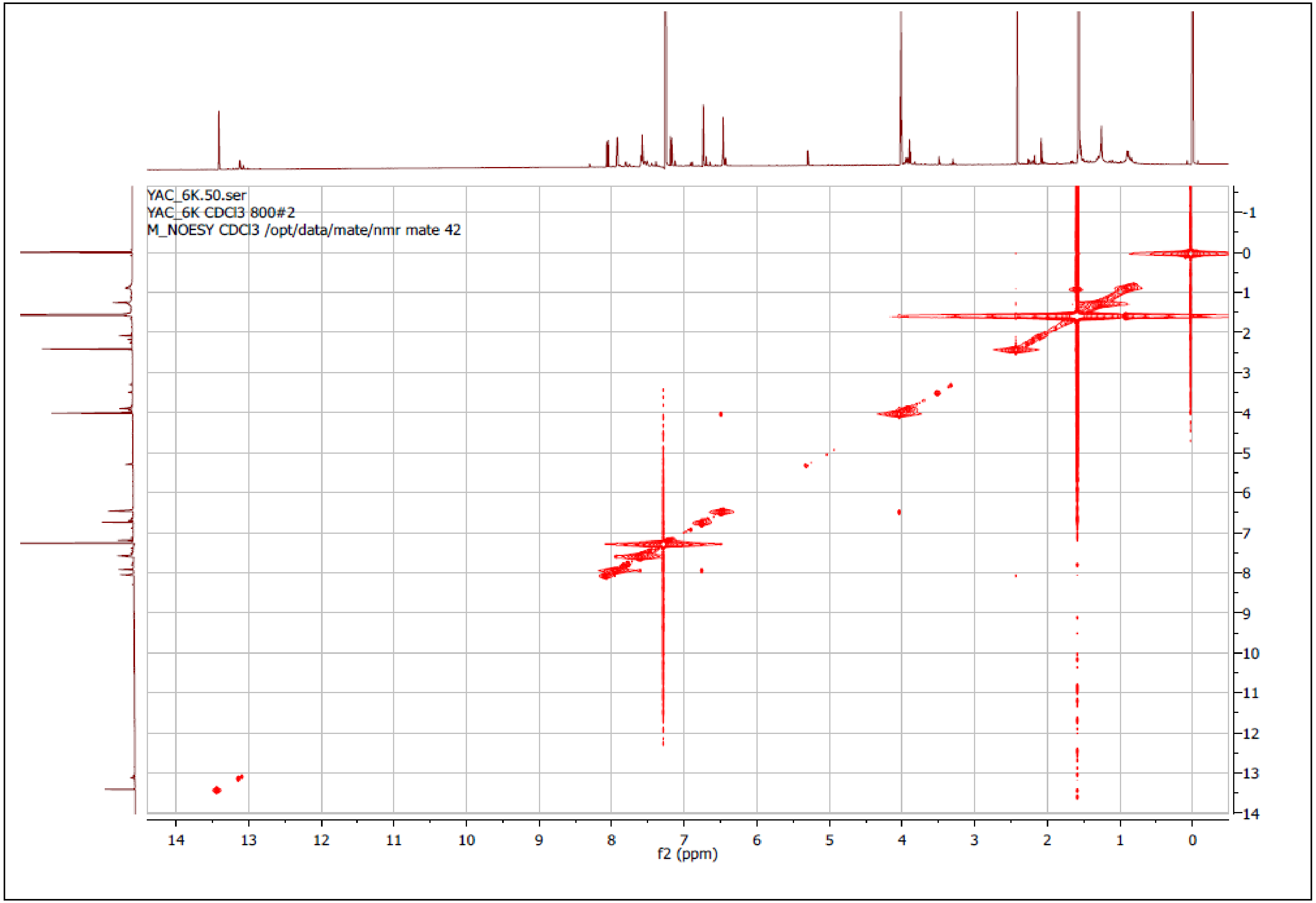

Fig. S11: NOESY spectrum of compound **2** ($CDCl_3$)

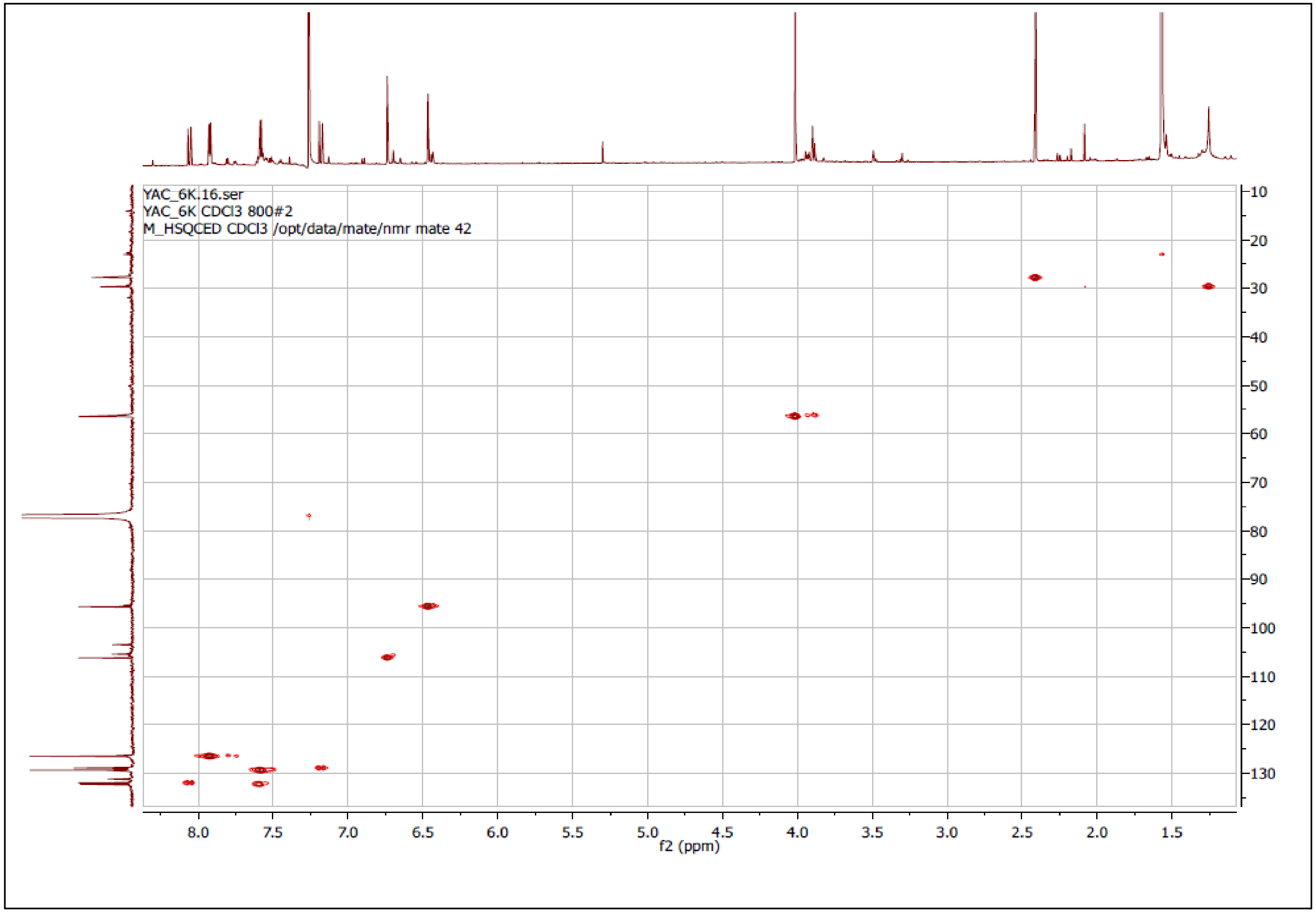

Fig. S12: HSQC spectrum of compound **2** ($CDCl_3$)

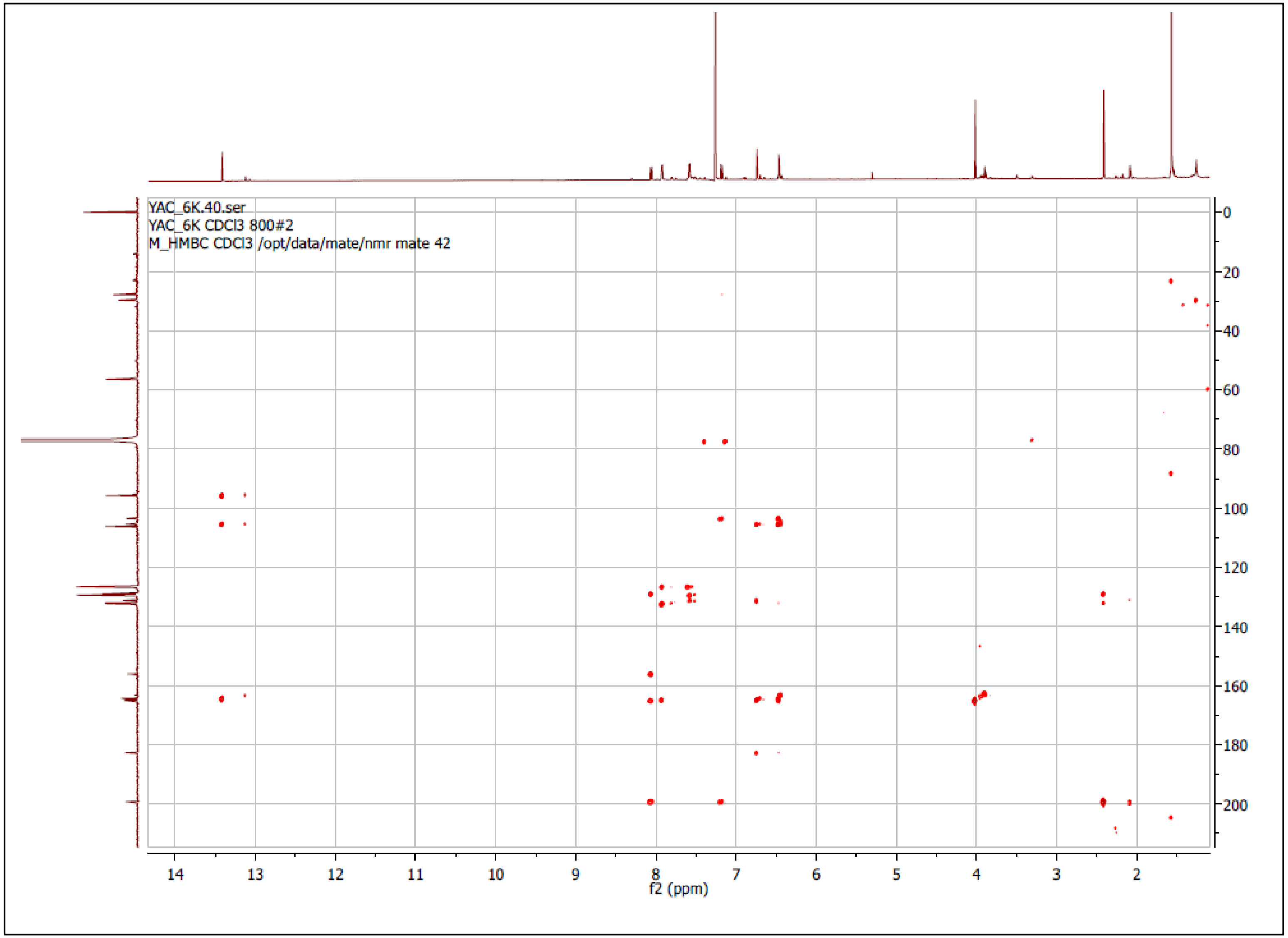

Fig. S13: HMBC spectrum of compound **2** ($CDCl_3$)

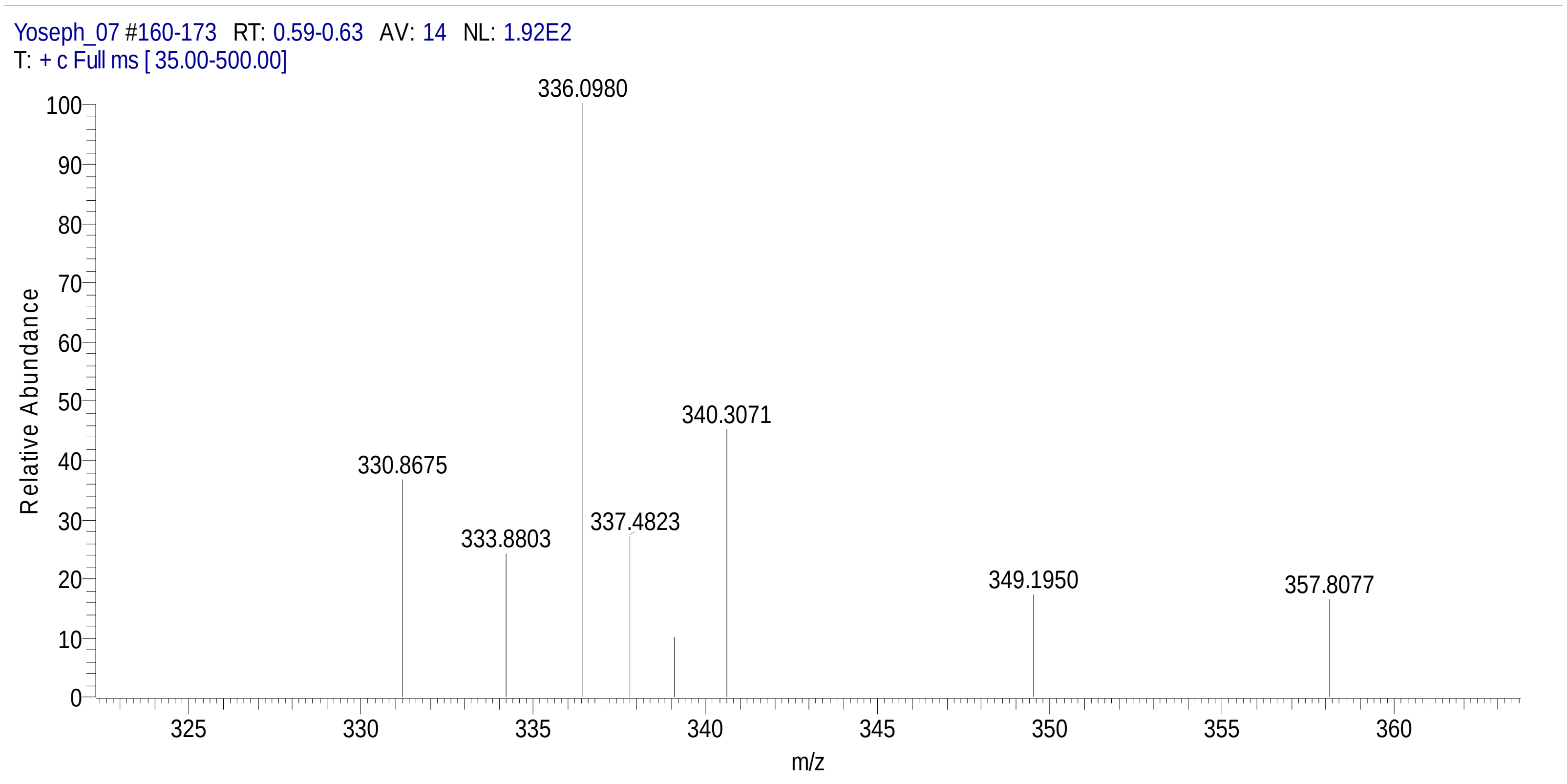

Fig. S14: HRMS of compound **2**

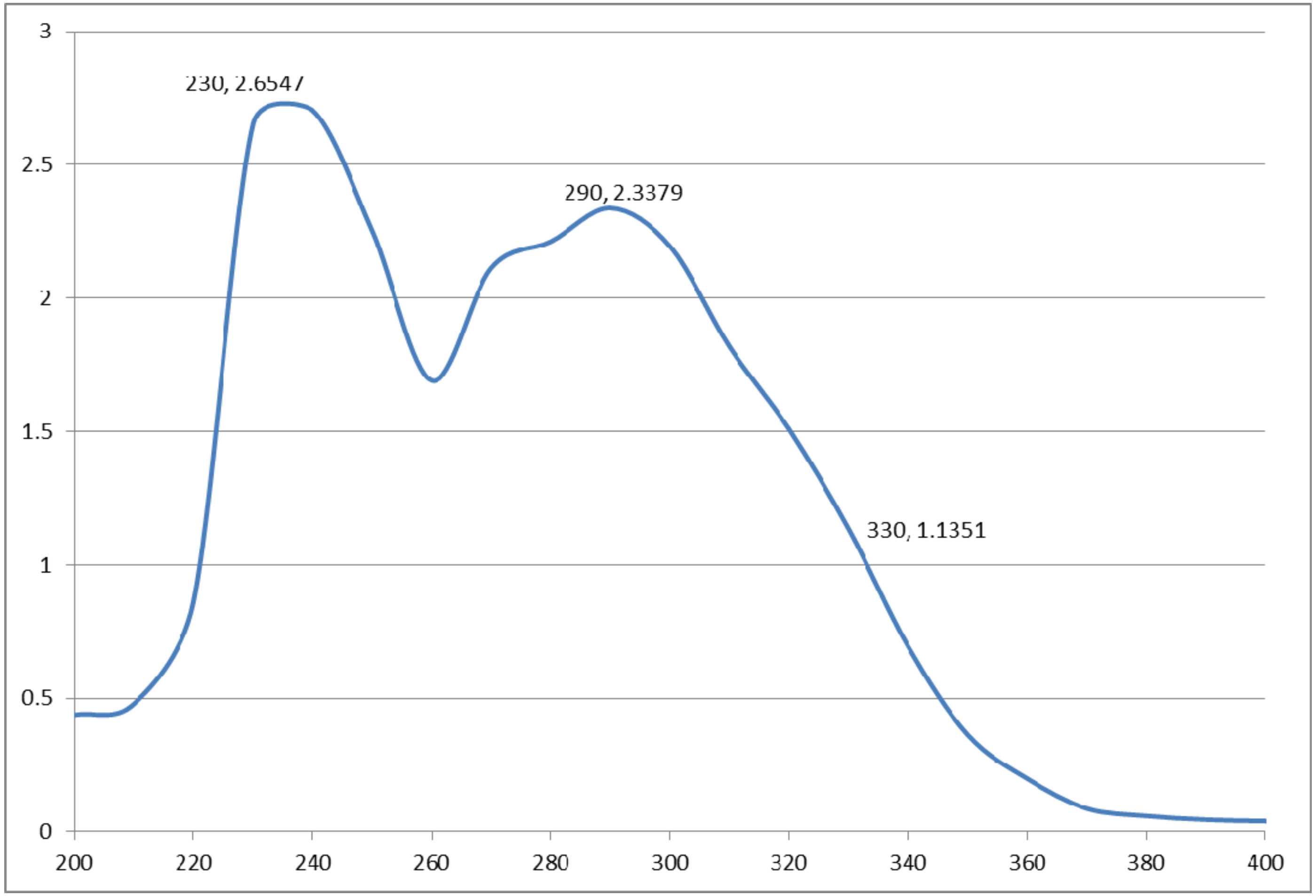

Fig. S15: UV spectrum of compound **2**

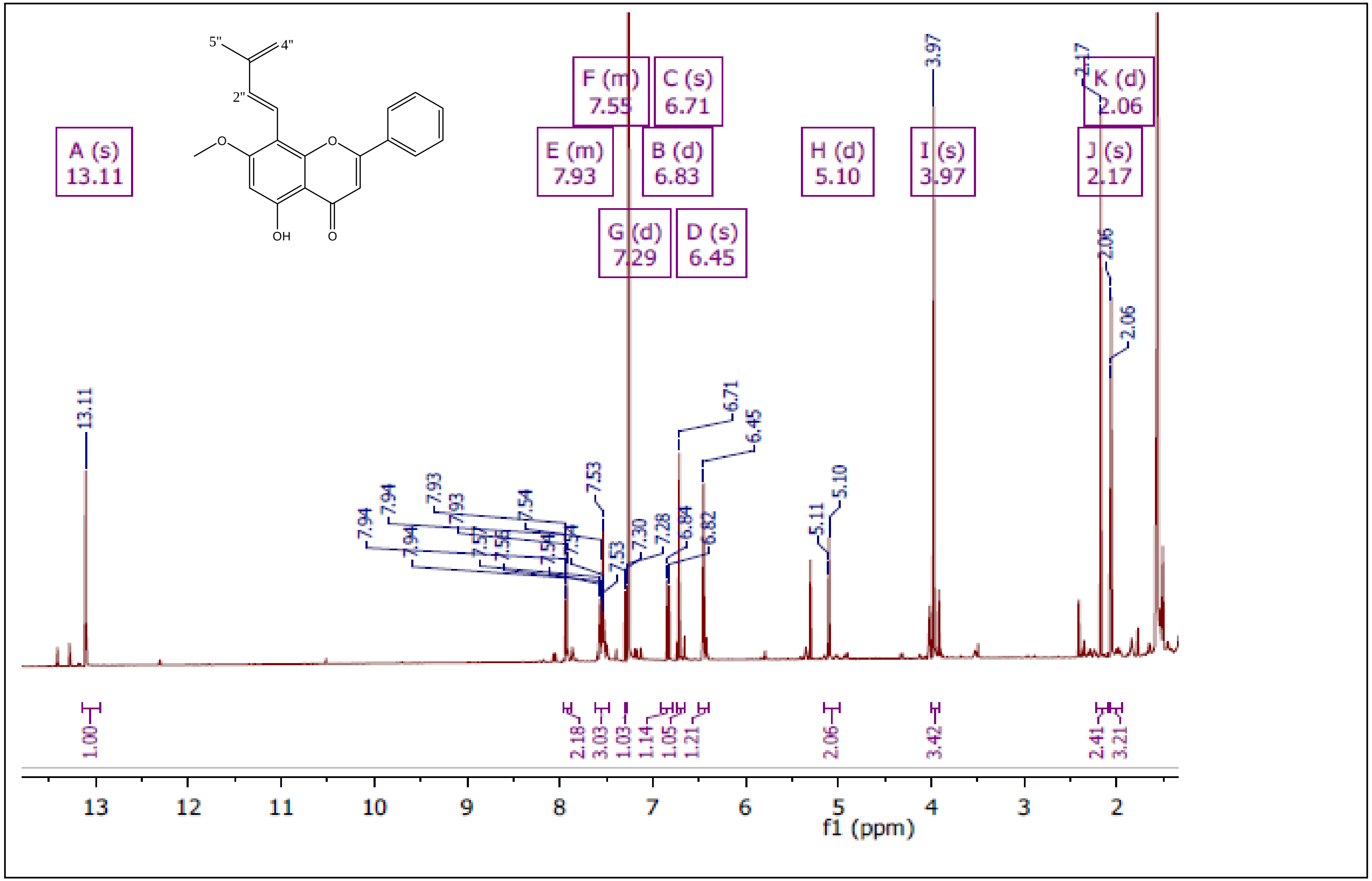

Fig. S16: ^{1}H NMR spectrum of compound **3** (800 MHz; $CDCl_3$)

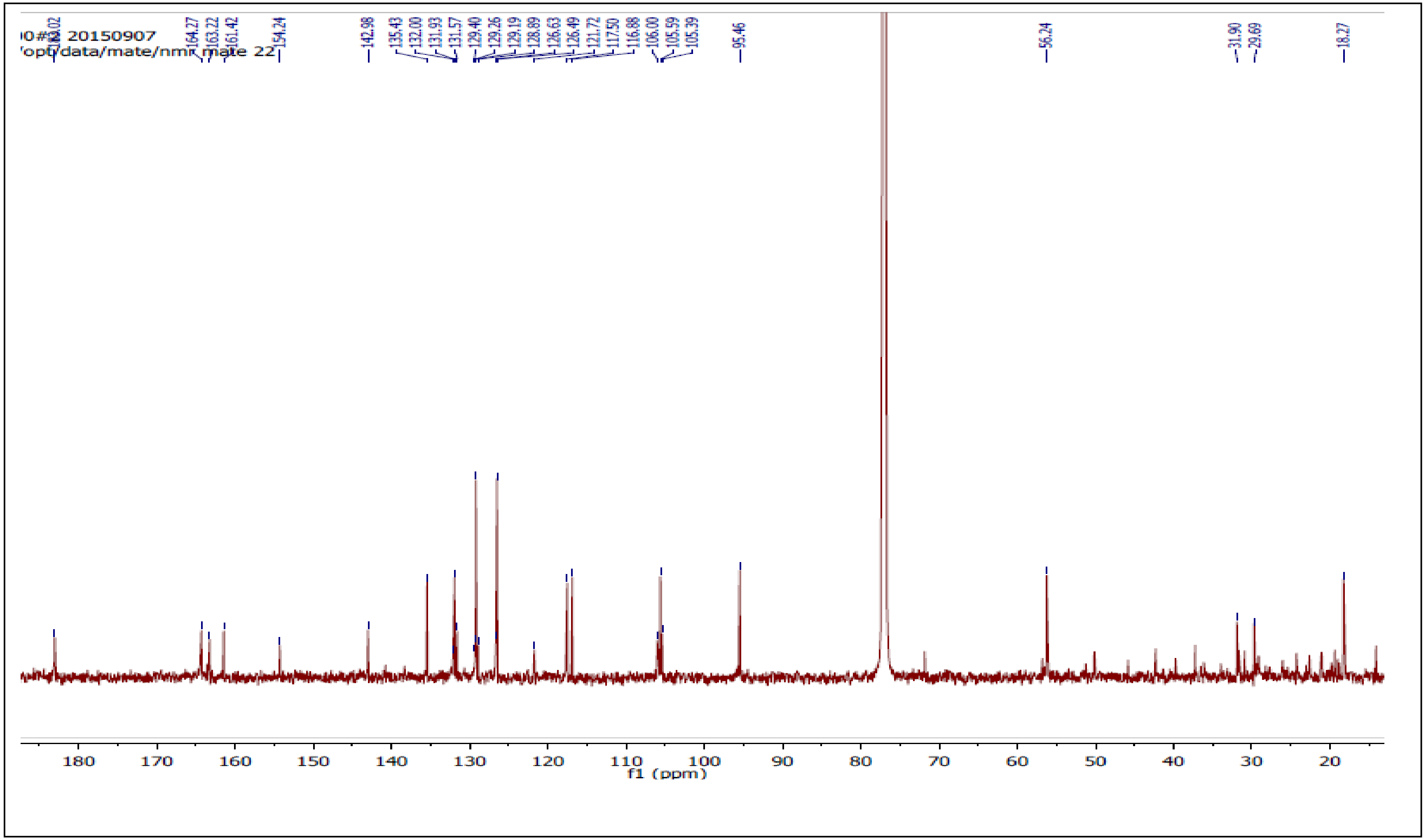

Fig. S17: ^{13}C NMR spectrum of compound **3** (200 MHz; $CDCl_3$)

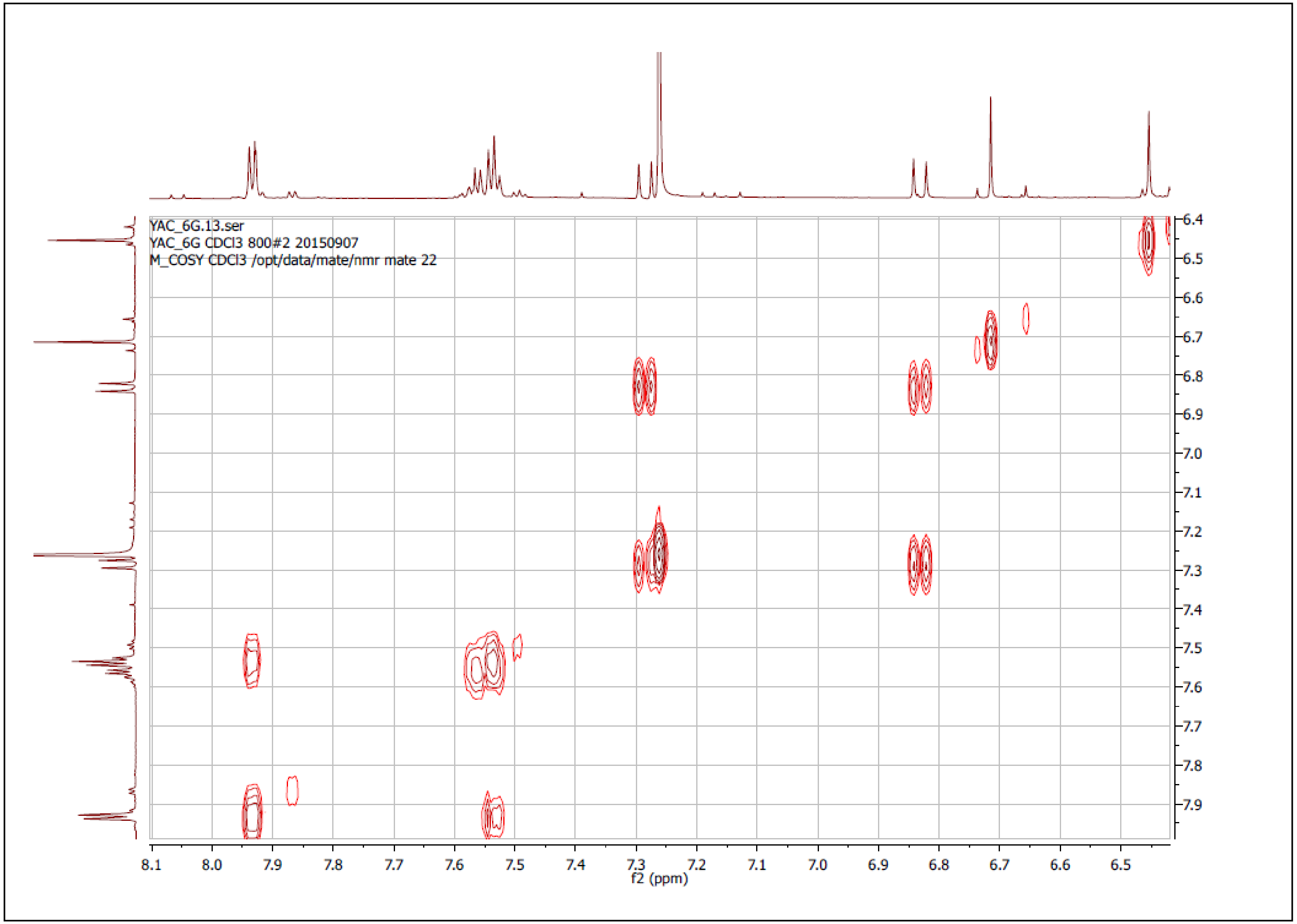

Fig. S18: COSY NMR spectrum of compound **3** ($CDCl_3$)

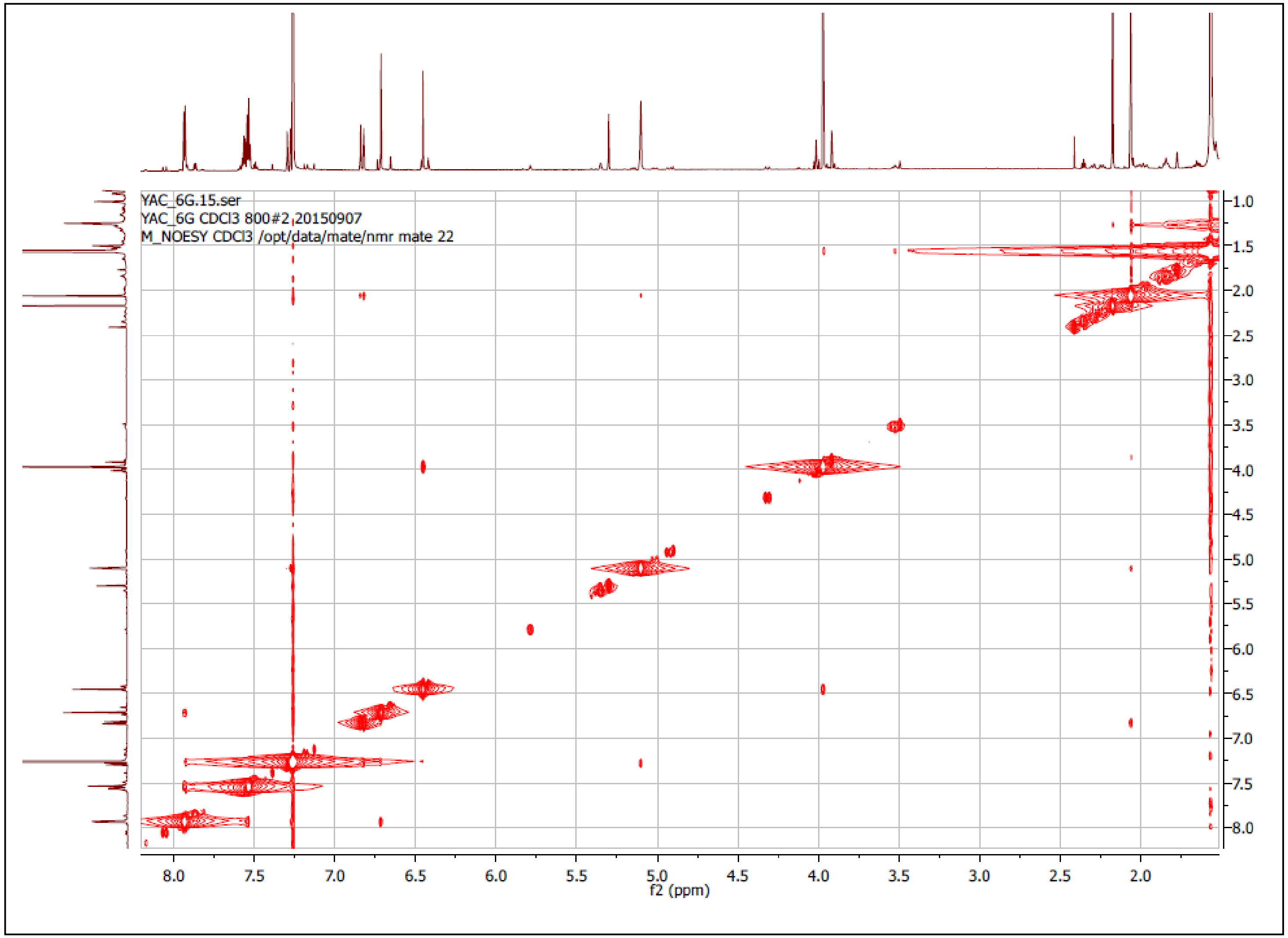

Fig. S19: NOESY spectrum of compound **3** ($CDCl_3$)

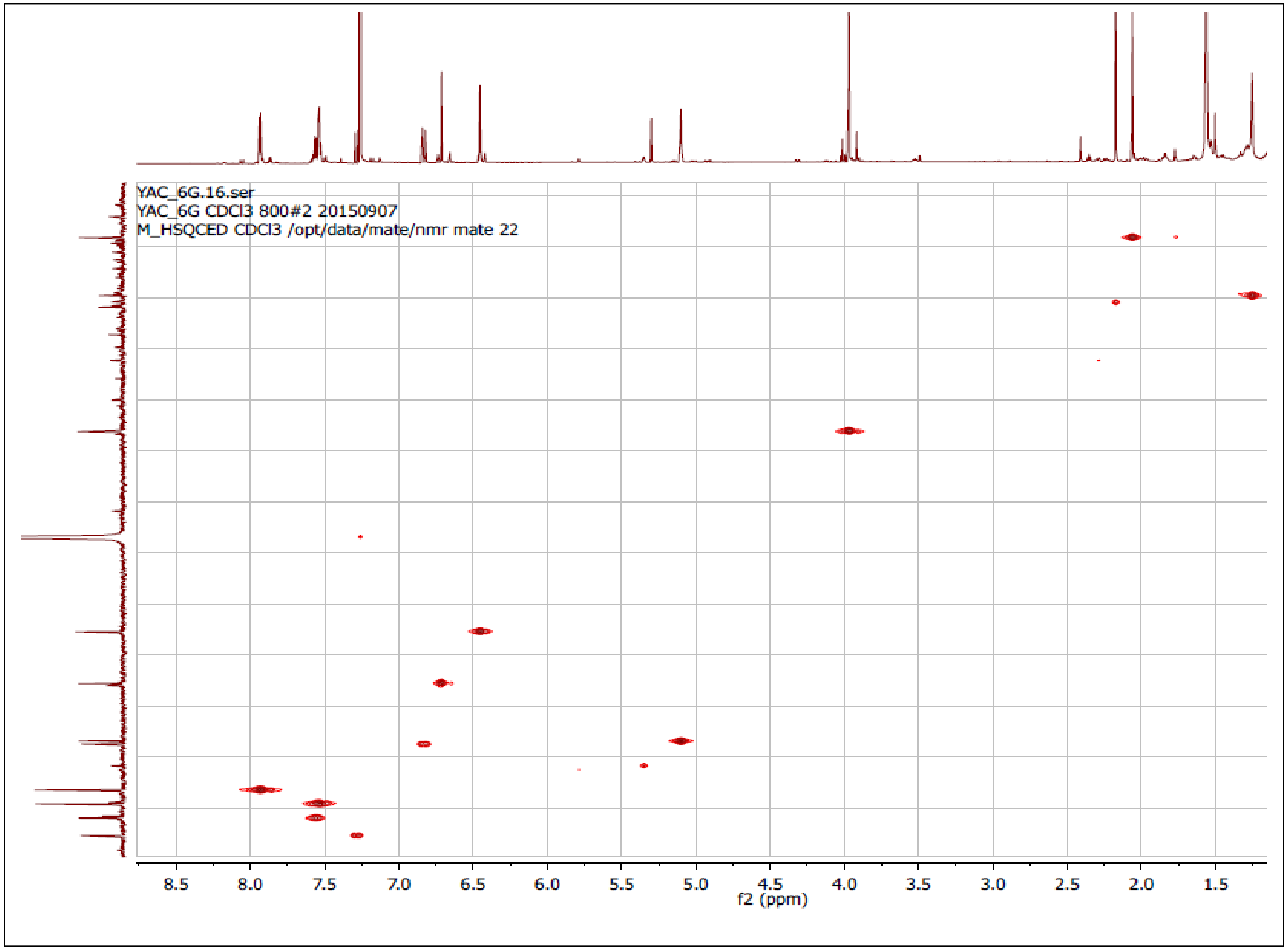

Fig. S20: HSQC NMR spectrum of compound **3** ($CDCl_3$)

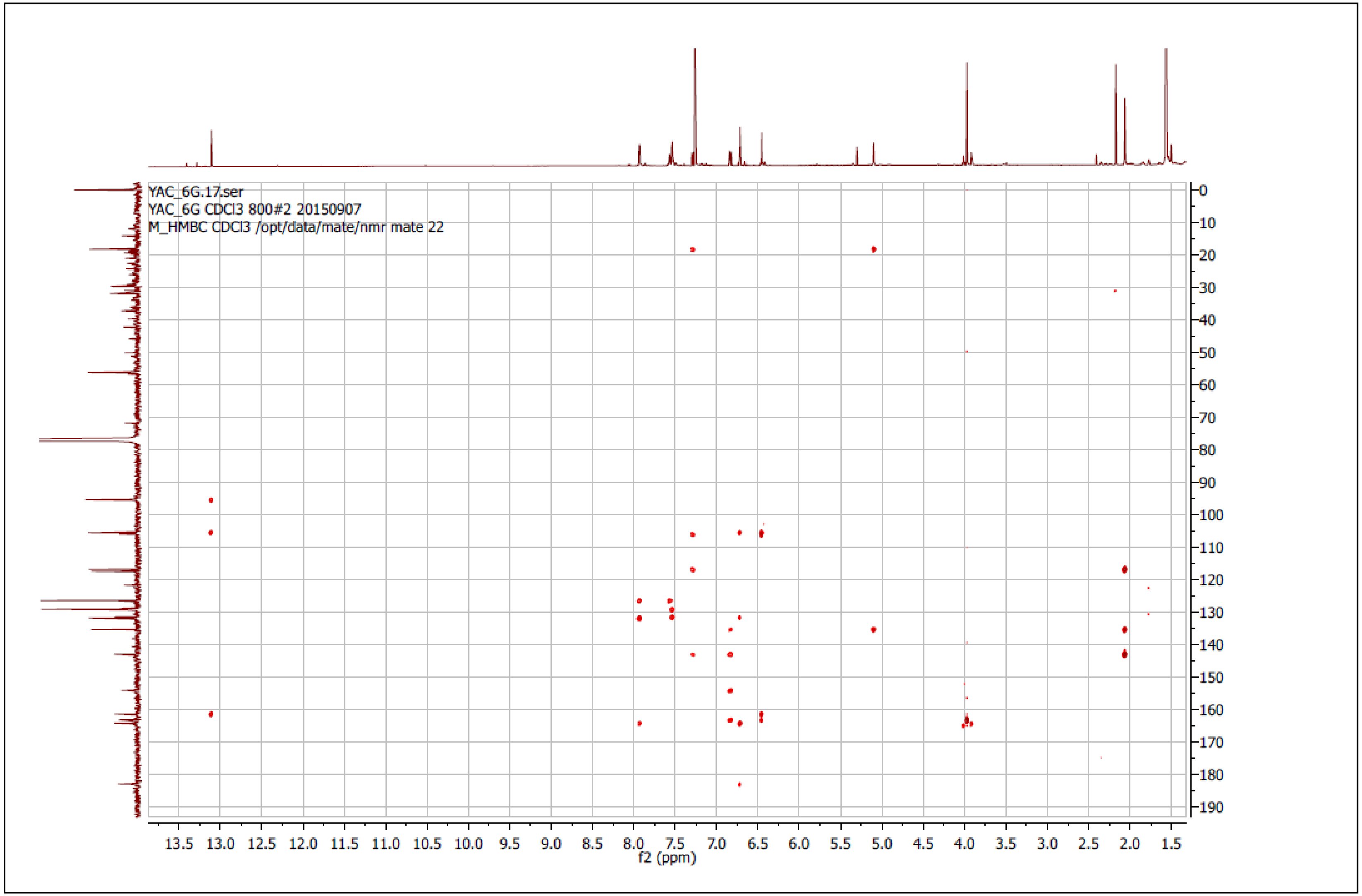

Fig. S21: HBMC spectrum of compound **3** ($CDCl_3$)

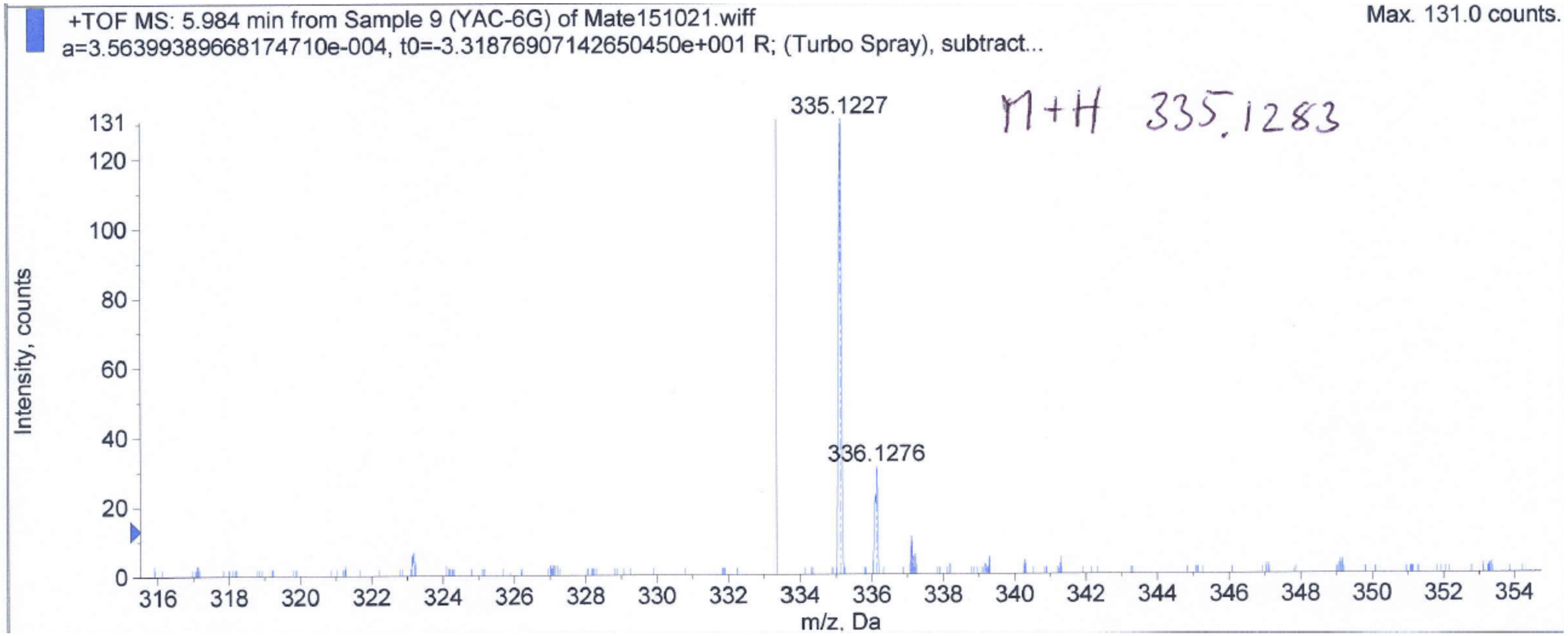

Fig. S22: HRMS of compound **3**

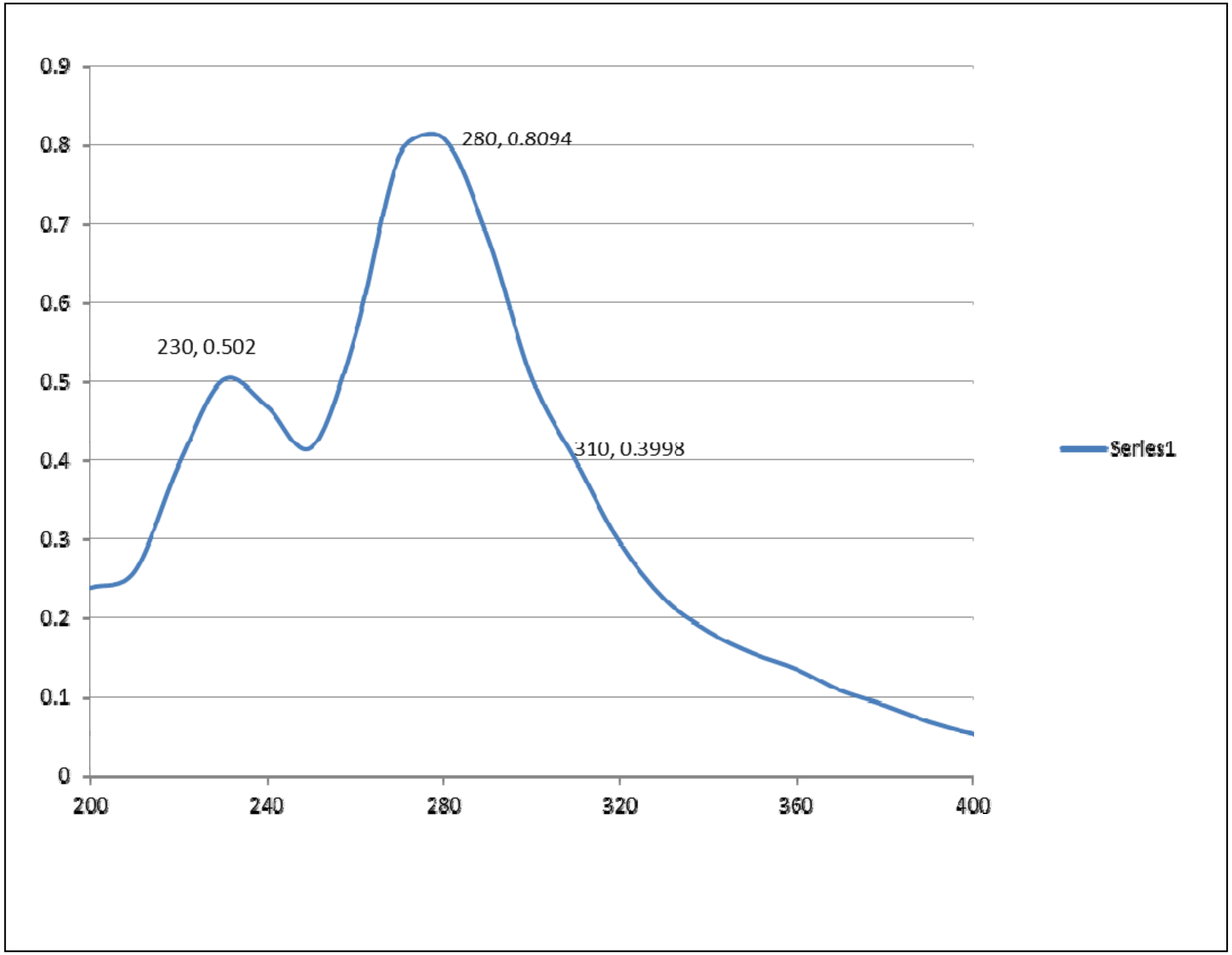

Fig. S23: UV spectrum of compound **3**

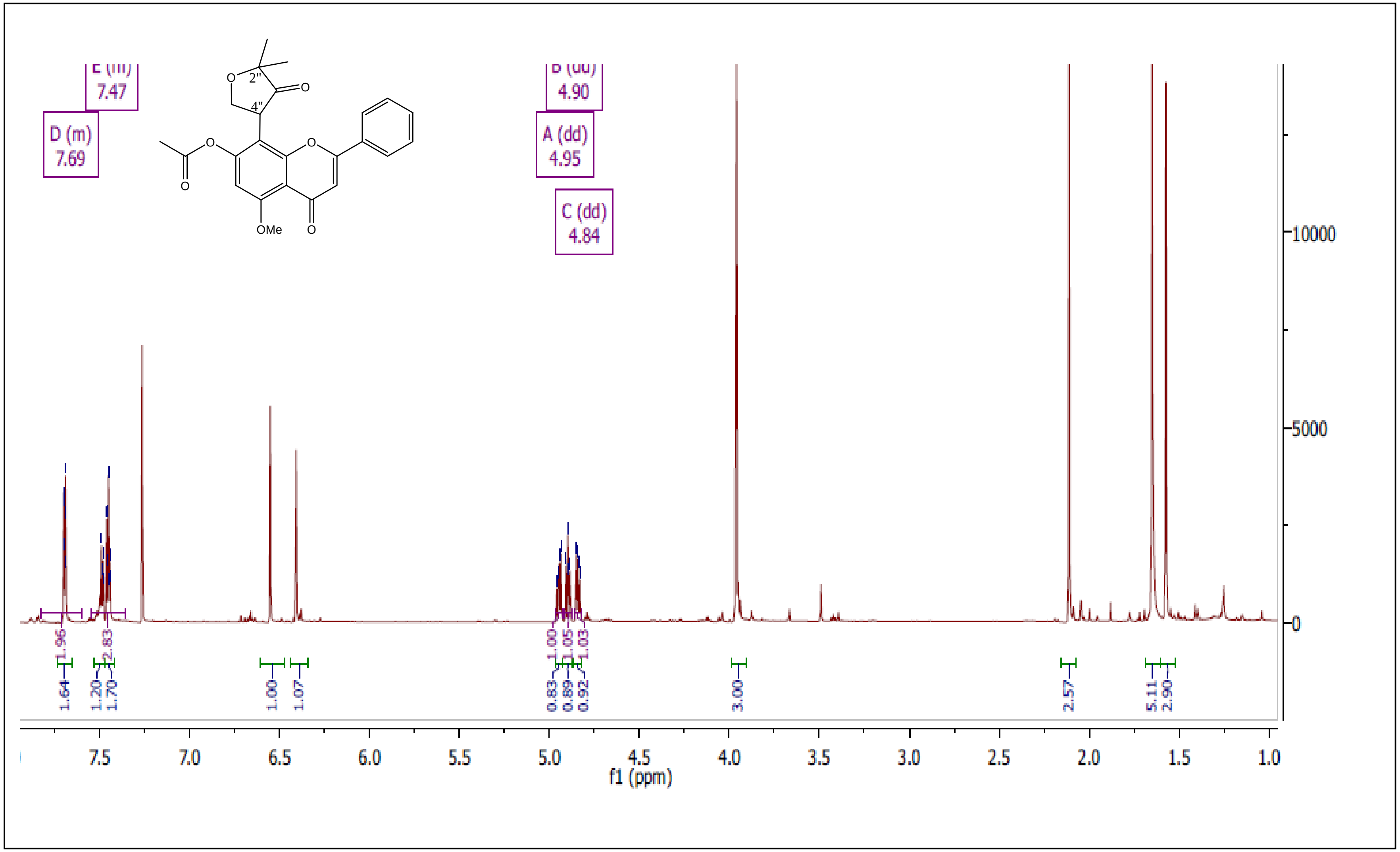

Fig. S24: ^{1}H NMR spectrum of compound **4** (800 MHz; $CDCl_3$)

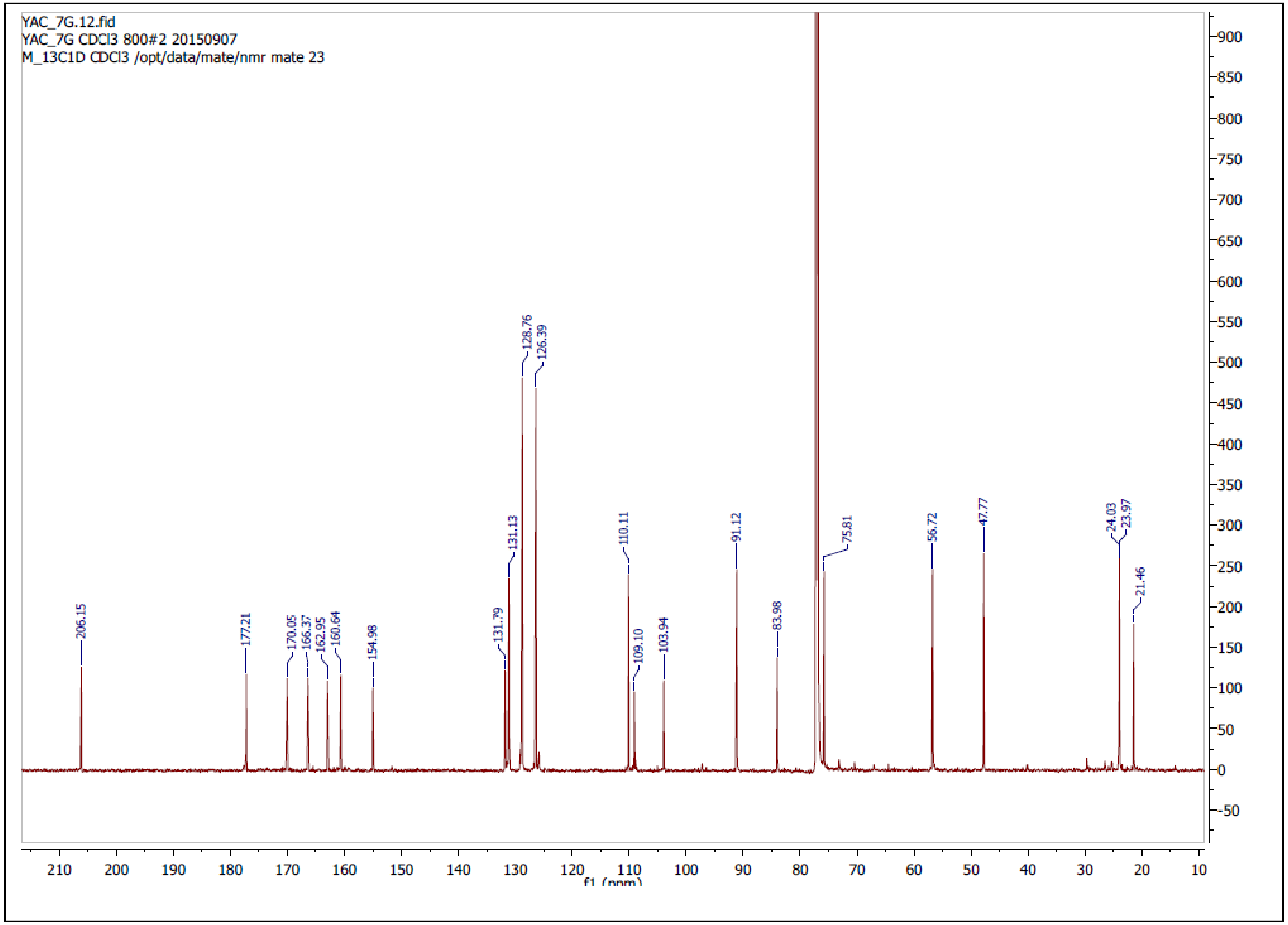

Fig. S25: ^{13}C NMR spectrum of compound **4** (200 MHz; $CDCl_3$)

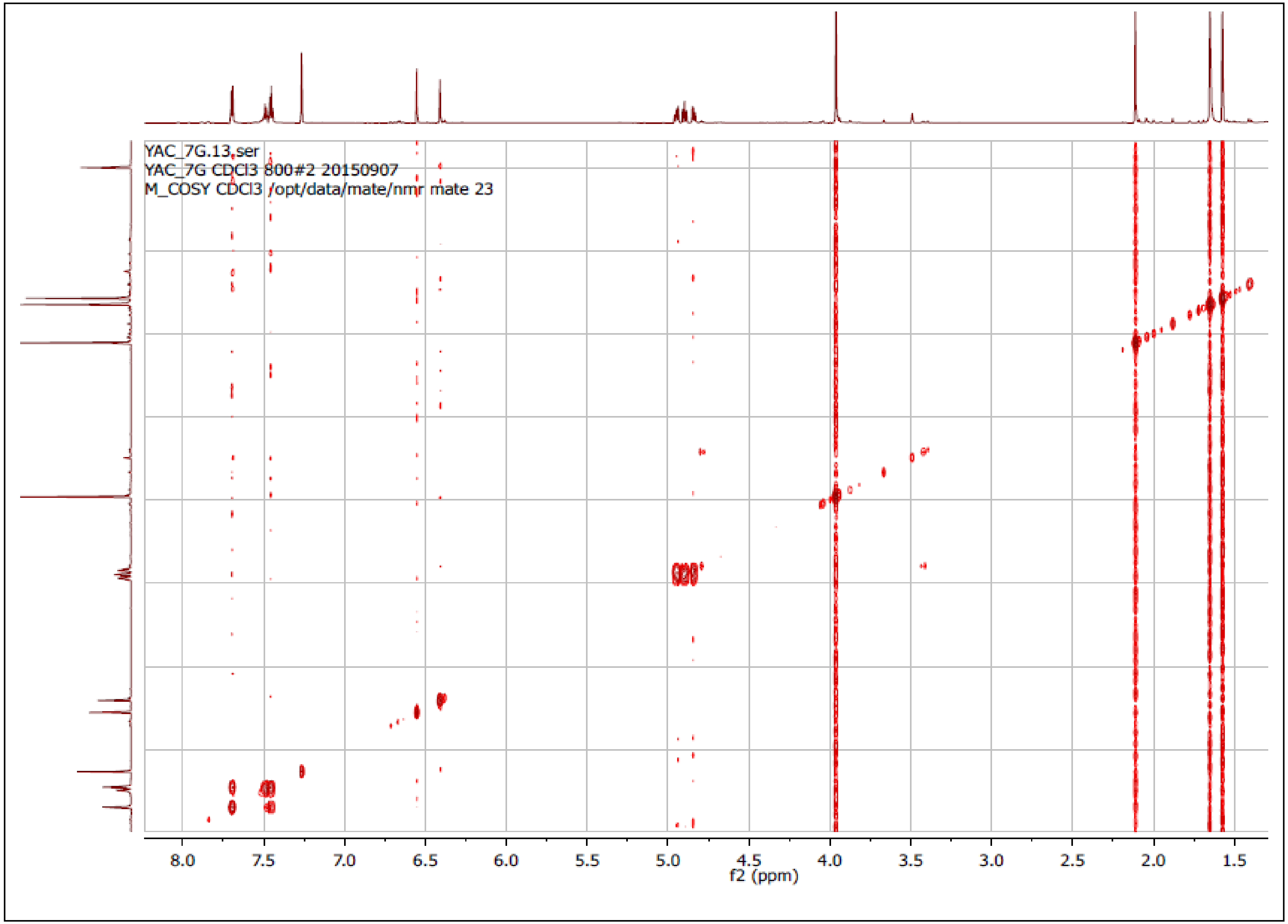

Fig. S26: COSY NMR spectrum of compound **4** ($CDCl_3$)

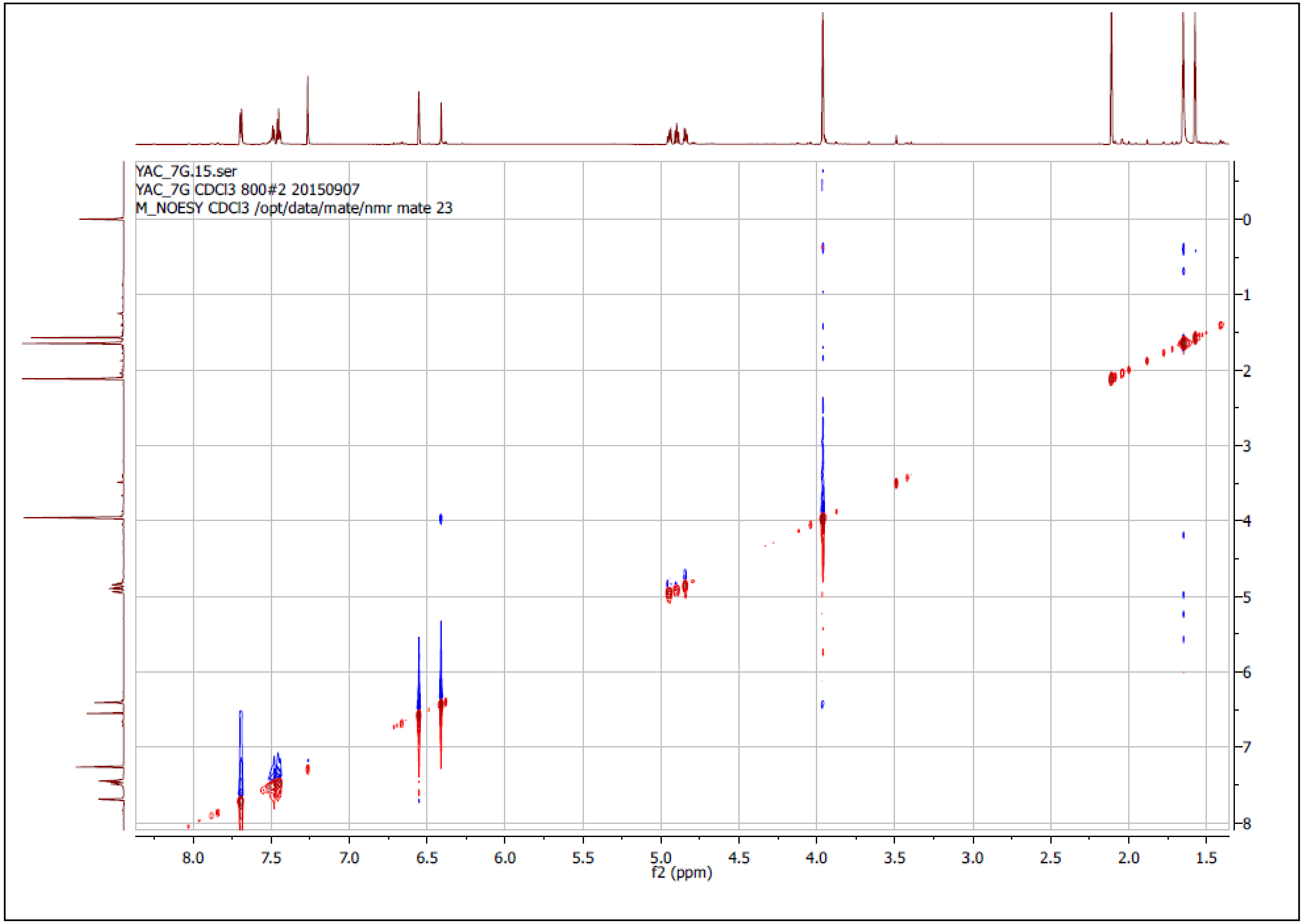

Fig. S27: NOESY spectrum of compound **4** ($CDCl_3$)

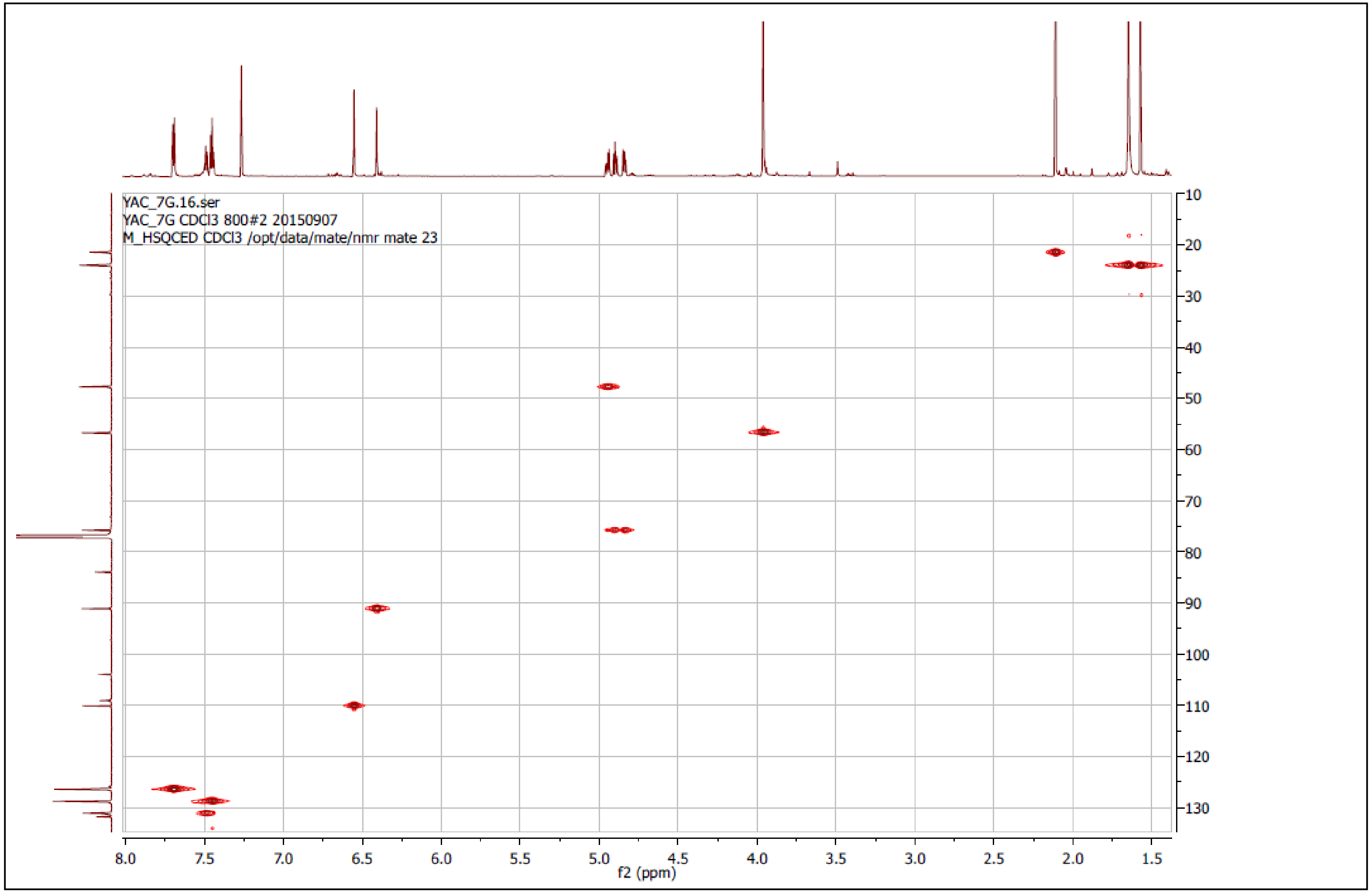

Fig. S28: HSQC NMR spectrum of compound **4** ($CDCl_3$)

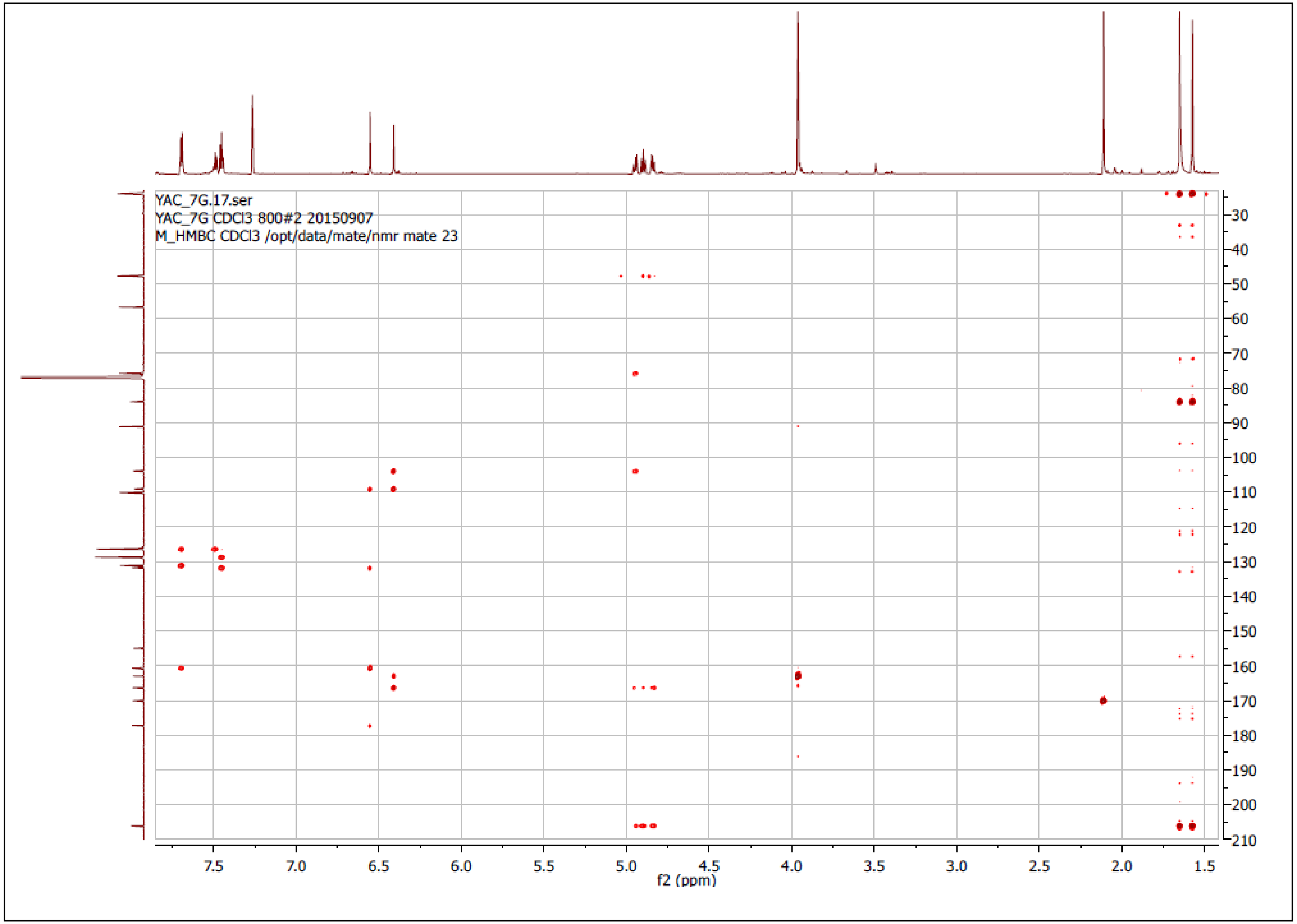

Fig. S29: HMBC NMR spectrum of compound **4** ($CDCl_3$)

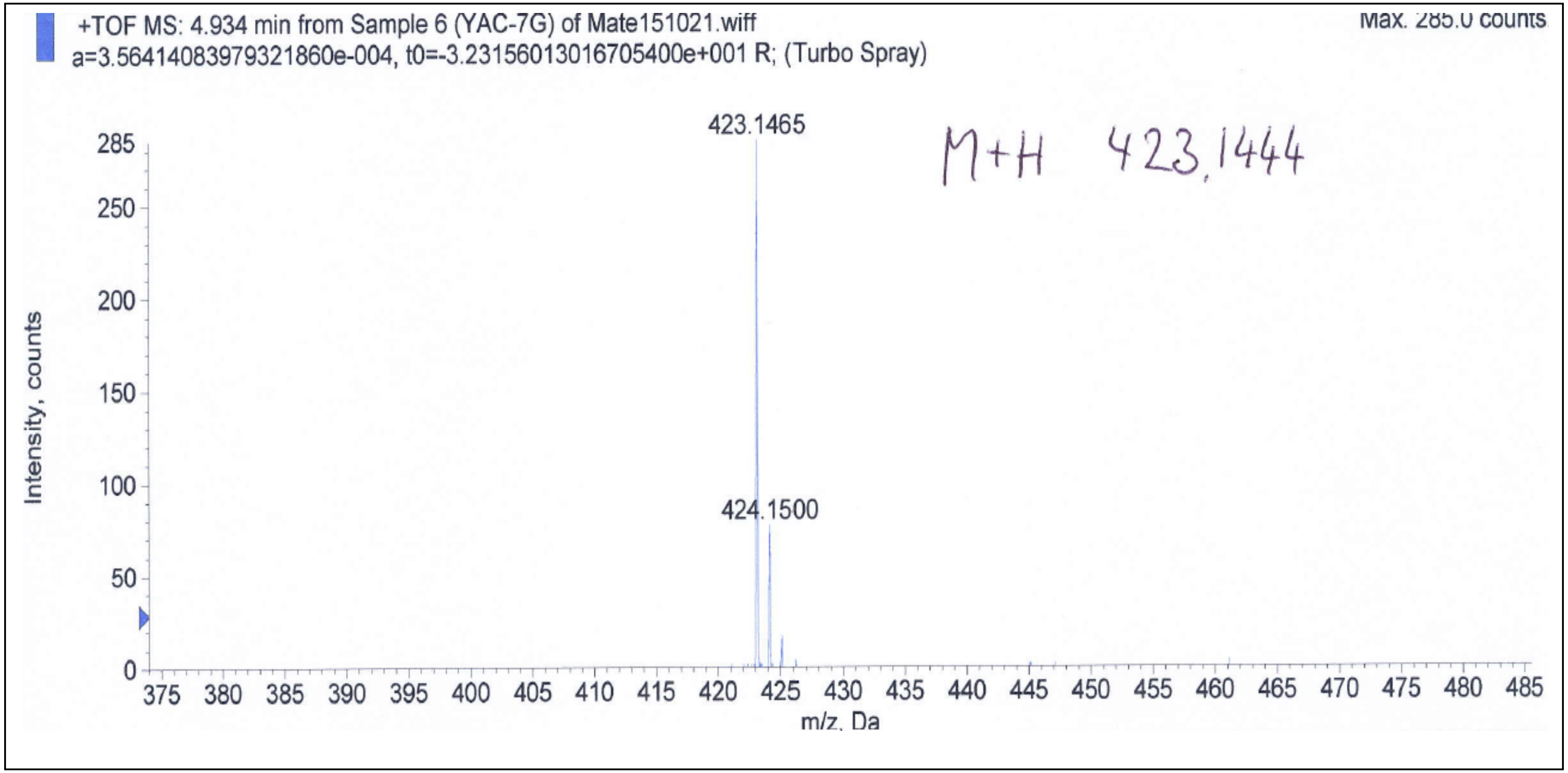

Fig. S30: HRMS of compound **4**

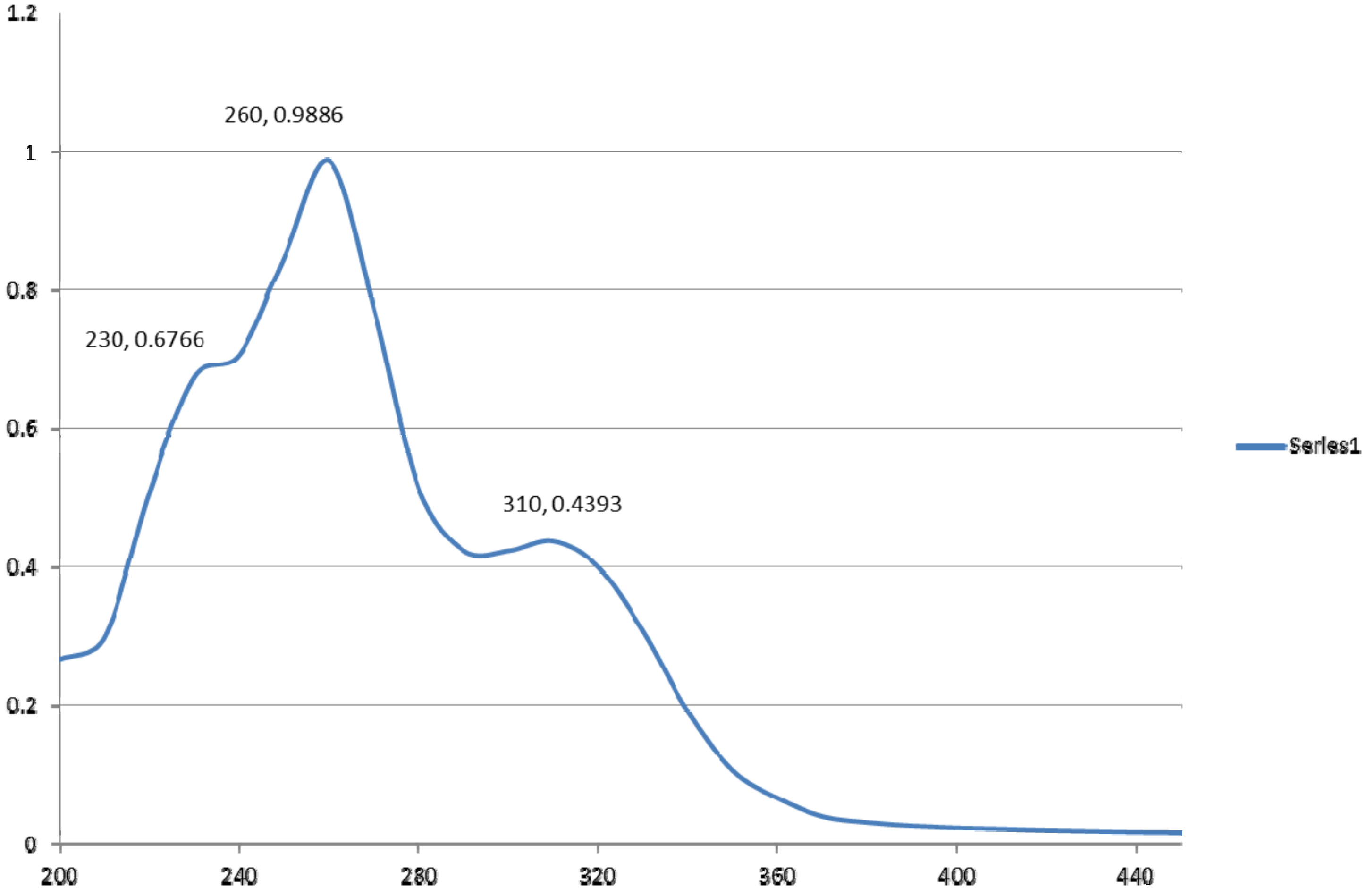

Fig. S31: UV spectrum of compound **4**

Table S1. ^{1}H (800 MHz) and ^{13}C (200 MHz) NMR spectroscopic data for compound **5**, $CDCl_3$

Position	δ_C (ppm)	δ_H (ppm), *m*	HMBC correlations
2	153.91	8.00 *s*	C-3, C-4, C-8a, C-1’
3	122.94		
4	180.77		
4a	104.97		
5	163.73		
5-OH		12.95 *s*	
6	99.21	6.26 *d (2.11)*	C-4a, C-5, C-7, C-8
7	165.12		
8	93.88	6.39 *d (2.11)*	C-4a, C-6, C-7, C-8a
8a	158.26		
1’	122.60		
2’,6’	130.0	7.41 *d (8.65)*	C-3, C-4’, C- 2’, C-6’
3’,5’	115.07	6.90 *d (8.65)*	C-1’, C-3’, C-4’, C-5’
4’	157.05		

Table S2. ^{1}H (800 MHz) and ^{13}C (200 MHz) NMR spectroscopic data for compound **6**, $CDCl_3$

**Position**			
	δ_C (ppm)	δ_H (ppm), *m*, (*J* in Hz)	HMBC correlations
**2**	78.98	5.42 *dd* (3.04, 13.03)	C-4,C-1',C-2'/6', C-8a
**3**	43.47	3.05 *dd* (13.00, 17.10)<br>2.85 *dd*(3.09, 17.12)	C-2, C-4, C-1'<br>C-4, C-4a, C-1'
**4(C=O)**	196.20		
**4a**	103.19		
**5**	162.23		
**5(OH)**		11.99 *s*	C-5, C-6, C-4a
**6**	96.92	6.03 *s*	C-4a, C-5, C-7, C-8
**7**	163.72		
**8**	109.02		
**8a**	159.61		
**1'**	138.68	**-**	
**2'/6'**	125.93	7.46	C-2, C-2'/6', C-3'/5'
**3'/5'**	128.79	7.45	C-1', C-3'/5'
**4'**	128.57	7.39	C-2'/6'
**1''**	21.80	3.33 *bt*	C-7, C-8, C-8a, C-3'', C-2''
**2''**	121.56	5.22 *btt*	C-1'', 3''-Me_2
**3''**	134.97		
**3''-Me_2**	17.84<br>25.82	1.73<br>1.73	C-3'', C-2''

Table S3. ^{1}H (800 MHz) and ^{13}C (200 MHz) NMR spectroscopic data for compound **7**, $CDCl_3$

**Position**			
	δ_C (ppm)	δ_H (ppm), *m*, (*J* in Hz)	HMBC correlations
**1**			
**2**	78.95	5.42 *dd* (13.17, 2.39)	
3	45.64	2.83 *dd* (16.46, 2.98)<br>2.99 *dd* (16.49, 13.16)	C-2, C-4, C-1'<br>C-4, C-4a, C-1'
**4(C=O)**	176.94		
**4a**	105.66		
**5**	162.13		
**6**	93.79	6.06 *s*	C-4, C-4a, C-5, C-7, C-8
**7**	160.01		
**8**	102.89		
**8a**	158.79		
**1'**	138.95	-	
**2'/6'**	125.93	7.46	C-2, C-2'/6', C-3'/5', C-4'
**3'/5'**	128.73	7.42	C-3'/5', C-1'
**4'**	128.90	7.38	C-2'/6'
**2''**	78.03		
**3''**	126.32	5.47 *d* (10.0)	C-8, C-2'', 2''-Me_2
**4''**	115.99	6.60 *d* (10.0)	C-7, C-8, C-8a, C-2''
2''-Me_2	28.20<br>28.50	1.45 *s*<br>1.46 *s*	C-3'', C-2'', 2''-Me_2
**OMe (C-5)**	56.20	3.90 *s*	C-5

Table S4. ^{1}H (800 MHz) and ^{13}C (200 MHz) NMR spectroscopic data for compound **8**, $CDCl_3$

Position	δ_C (ppm)	δ_H (ppm), *m*	HMBC correlations
2	153.91	8.00 *s*	C-3, C-4, C-8a, C-1’
3	122.94		
4	180.77		
4a	104.97		
5	163.73		
5-OH		12.95 *s*	
6	99.21	6.26 *d (2.11)*	C-4a, C-5, C-7, C-8
7	165.12		
8	93.88	6.39 *d (2.11)*	C-4a, C-6, C-7, C-8a
8a	158.26		
1’	122.60		
2’,6’	130.0	7.41 *d (8.65)*	C-3, C-4’, C- 2’, C-6’
3’,5’	115.07	6.90 *d (8.65)*	C-1’, C-3’, C-4’, C-5’
4’	157.05		

Table S5. ^{1}H (800 MHz) and ^{13}C (200 MHz) NMR spectroscopic data for compound **9**, $CDCl_3$

Position	δ_C (ppm)	δ_H (ppm), *m*	HMBC correlations
2	161.07		
3	108.86	6.63 *s*	C-2, C-4, C-4a, C-1′
4	204.51		
4a	109.10		
5	161.23		
6	91.63	6.45 *s*	C-4, C-4a, C-5, C-7, C-8
7	161.45		
8	98.30		
8a	156.75		
1′	131.84		
2′,6′	126.21	7.71 *m*	C-2, C-4′, C- 2′, C-6′
3′,5′	128.74	7.41 *m*	C-1′, C-3′, C-5′
4′	131.11	7.45 *m*	C-2′, C-6′
2''	88.07		
3′′	204.51		
4′′	109.51		
5''	175.67	6.31, *s*	C-2″, C-3″, C-4″
2 x Me-2''	23.05	1.57, *s*	C-3″, C-2″

Table S6. ^{1}H (800 MHz) and ^{13}C (200 MHz) NMR spectroscopic data for compound **10**, $CDCl_3$

**Position**			
	δ_C (ppm)	δ_H (ppm), *m*, (*J* in Hz)	HMBC correlations
**1**		-	
**2**	158.39		
3	135.07		
**4(C=O)**	178.36		
**4a**	106.16		
**5**	161.59		
**5(OH)**		12.61 *s*	**C-4a, C-6, C-5**
**6**	94.19	6.74 *d* (2.20)	**C-4a, C-6, C-8a, C-7**
**7**	162.13		
**8**	99.14	6.48 *d* (2.20)	**C-6, C-4a, C-7**
**8a**	156.67		
**1'**	120.98	-	
**2'/6'**	130.59	7.81 *d* (8.69)	C-**3'/5', C-2'/6', C-4'**
**3'/5'**	115.18	6.96 *d* (8.21)	C-**3'/5', C-1', C-4'**
**4'**	160.36	-	
**2''**	98.44	5.58 *s*	**C-7**
**3''**	70.26	3.86 *bd*	
**4''**	71.13	3.16 *m*	
**5''**	72.04	3.33 *t*	
**6''**	70.54	3.44 *m*	
**2'''**	102.10	5.42 s	**C-3**
**3'''**	70.53	4.00 *bd*	
**4'''**	70.78	3.50 *bd*	
**5'''**	70.67	3.66 *bd*	
**6'''**	71.55	3.15 *m*	
**Me(6'')**	18.36	1.15 *d*	
**Me (6''')**	17.95	0.82 *d*	

Table S7. ^{1}H (800 MHz) and ^{13}C (200 MHz) NMR spectroscopic data for compound **11**, $CDCl_3$

**Position**			
	δ_C (ppm)	δ_H (ppm), *m*, (*J* in Hz)	HMBC correlations
**1**	70.96	2.59 *d* (2.26)	
**2**	69.25	2.44 *dd* (2.25, 9.73)	C-5
**3**	69.78	2.39 *dd* (2.24, 9.70)	
**4**	71.54	2.28 *t* (9.46, 9.46)	C-3, C-5,
**5**	82.14	1.95 *t* (9.46, 9.46)	C-3, C-4, OMe-3,
**6**	70.66	2.59 *d* (2.26)	
**OMe-5**	58.10	2.31	
